# Teledermatology vs. Face-to-Face Dermatology for the Diagnosis of Melanoma: A Systematic Review

**DOI:** 10.3390/cancers17172836

**Published:** 2025-08-29

**Authors:** María López-Pardo Rico, Manuel Ginarte Val, María Dolores Sánchez-Aguilar Rojas, Lorena Martínez Leboráns, Carmen Rodríguez Otero, Ángeles Flórez

**Affiliations:** 1 Dermatology Department, Hospital Universitario Lucus Augusti, 27003 Lugo, Spain; mlopezpardorico@gmail.com; 2 Santiago de Compostela University, 15705 Santiago, Spain; lolasaguilar@gmail.com (M.D.S.-A.R.); angeles.florez.menendez@sergas.es (Á.F.); 3 Dermatology Department, Complexo Hospitalario Universitario de Santiago de Compostela, 15706 Santiago, Spain; lorena.martinez.leborans@sergas.es; 4 Library, Complexo Hospitalario Universitario de Ourense, 32005 Ourense, Spain; maria.carmen.rodriguez.otero@sergas.es; 5 Bibliosaúde—Biblioteca Virtual del Sistema Públido de Salud de Galicia, Sergas, Spain

**Keywords:** teledermatology, teledermoscopy, melanoma, smartphone app, artificial intelligence, early diagnosis, diagnostic accuracy, healthcare access, healthcare efficiency

## Abstract

Background: Cutaneous melanoma is the leading cause of skin cancer-related mortality, and early detection is crucial to improving prognosis. Teledermatology (TD) has increasingly been adopted in melanoma care to address growing demand and limited access to specialists. This systematic review aimed to evaluate the role of TD in the diagnosis and management of suspected melanoma under real-world clinical conditions. Methods: The review was conducted and reported in accordance with the PRISMA 2020 guidelines. Literature searches were performed in PubMed, Embase, Web of Science, and Scopus up to December 2024. Studies assessing TD use in routine practice were included, focusing on diagnostic accuracy, prognostic indicators, waiting times, satisfaction, and economic outcomes. Results: TD showed high sensitivity and specificity, particularly when dermoscopic images and expert interpretation were available. Some studies reported reduced Breslow thickness and shorter delays compared to face-to-face care. Overall satisfaction was high among both patients and clinicians. Economic evaluations suggested potential cost savings, although formal analyses were limited. The use of artificial intelligence tools yielded mixed results and was generally perceived with caution in the absence of clinical supervision. Conclusions: Teledermatology appears to be a useful approach for improving access and supporting early diagnosis in melanoma care. Its effectiveness depends on proper implementation and integration into clinical workflows.

## Simple Summary

Cutaneous melanoma remains the leading cause of skin cancer-related mortality, with early detection being essential to improving prognosis. Teledermatology, which allows clinicians to assess skin lesions remotely using digital images, has gained relevance as a tool to support timely diagnosis, especially in the context of increasing demand and limited access to dermatologists. This review explored whether teledermatology improves care for patients with suspected melanoma in real-world clinical practice. We examined its diagnostic accuracy, impact on waiting times, satisfaction, and healthcare economic outcomes. Our findings suggest that teledermatology may be a valuable strategy to optimise access and streamline referrals when integrated effectively into existing care systems.

## 1. Introduction

Cutaneous melanoma remains the leading cause of skin cancer-related mortality, with early detection being essential to improving prognosis [1,2,3]. According to GLOBOCAN 2022, cutaneous melanoma accounted for approximately 331,722 new cases and 58,667 deaths globally, ranking 17th in incidence and 22nd in cancer-related mortality across all tumour types. Five-year prevalence reached over 1.26 million cases, highlighting the increasing burden of disease [4]. In 2020, Europe accounted for 50% of melanoma diagnoses and 45% of melanoma-related deaths worldwide. Melanoma is the sixth most commonly diagnosed cancer overall and the third most frequent malignancy among individuals aged 15–49, accounting for 8% of new diagnoses in this group [5]. Despite its high incidence, melanoma is no longer among the 15 leading causes of cancer mortality in Europe, reflecting the effectiveness of early detection and treatment [5,6,7,8,9]. However, projections for 2040 suggest a 20% increase in incidence among men, 8% among women, and a 27% rise in mortality [5,10], underscoring the urgency of optimising early diagnosis.

This rising incidence is driven primarily by two converging trends: increased cumulative ultraviolet (UV) radiation exposure, particularly among fair-skinned populations, and the progressive ageing of the European population—where one in five citizens is currently aged ≥65, and that proportion is expected to reach 25% by 2040 [5,11]. Given the age-associated risk of melanoma, this demographic shift will intensify demand for dermatological care. Simultaneously, many countries face a growing shortage of dermatologists, while heightened public awareness has led to a sustained rise in dermatology referrals [5]. Another contributing factor is diagnostic drift: advances in diagnostic tools, greater awareness campaigns, and more widespread use of opportunistic skin checks have increased the detection of early-stage or indolent melanomas that might previously have remained unnoticed. While this trend reflects improved vigilance, it also raises the possibility of overdiagnosis, with an increasing number of lesions being identified that may have limited biological significance [5]. Although population-based melanoma screening remains controversial [12] and is not widely implemented, there is a broad consensus that ensuring timely access to dermatologists is essential for effective triage and early-stage diagnosis [13].

In this context, teledermatology (TD)—defined as the use of telecommunication technologies to deliver dermatological care at a distance, most commonly through the exchange of digital images and clinical information [14]—has emerged as a practical solution to improve access, prioritisation, and resource use. TD facilitates early dermatological evaluation without requiring immediate in-person consultations, potentially reducing delays for high-risk lesions while alleviating pressure on specialist services. Although TD had been in use for years, the COVID-19 pandemic prompted a large-scale and rapid adoption of remote dermatological care. In 2020, over 75% of outpatient dermatology consultations were conducted remotely in some centres, and TD played a key role in the triage of suspicious lesions, avoiding unnecessary visits, and maintaining continuity of care during mobility restrictions [15].

Despite the expansion of TD and the increasing volume of related publications since 2020, no systematic review to date has specifically evaluated its role in improving access to dermatological care for patients with suspected or confirmed melanoma in routine clinical settings. Most existing reviews focus either on general dermatology or technological aspects, often excluding real-world implementation outcomes. The aim of this review is to synthesise current evidence on TD as a strategy to improve access and care pathways for melanoma. We specifically compare TD with conventional face-to-face (FTF) models regarding diagnostic accuracy, prognostic outcomes, waiting times, patient and clinician satisfaction, and health system efficiency, with emphasis on studies conducted in real-world contexts.

## 2. Materials and Methods

### 2.1. Study Design and Registration

This systematic review was reported in accordance with the PRISMA 2020 guidelines (Preferred Reporting Items for Systematic Reviews and Meta-Analyses) [16] and was prospectively registered in PROSPERO (registration number: 1068350). The objective was to assess the impact of TD, compared to traditional FTF referral, on access to dermatological care and diagnostic outcomes in adults with suspected cutaneous melanoma.

### 2.2. Eligibility Criteria

We included studies involving patients who underwent teledermatological evaluation—either store-and-forward, live video consultation, teledermoscopy, smartphone-based applications, or artificial intelligence tools—for suspected cutaneous melanoma. Acceptable comparators included conventional FTF dermatology referrals or the absence of intervention.

Eligible studies were required to report on at least one of the following outcomes: (i) diagnostic accuracy (sensitivity and/or specificity); (ii) Breslow thickness at diagnosis or AJCC staging (8th edition); (iii) time to dermatological assessment; (iv) patient or clinician satisfaction; or (v) economic outcomes. We included randomised controlled trials, prospective or retrospective observational studies, and cross-sectional designs.

We excluded studies that (i) did not report separate outcomes for melanoma when analysing mixed skin cancer populations; (ii) were case reports, editorials, commentaries, or conference abstracts without accessible full-text results; (iii) lacked quantitative data (e.g., qualitative-only designs); (iv) focused on non-cutaneous or metastatic melanoma; or (v) were based on image datasets without real-world clinical application.

### 2.3. Information Sources and Search Strategy

We systematically searched PubMed, Embase (via Ovid), Web of Science, and Scopus. The initial search was conducted on 10 December 2024 and updated on 20 December 2024 by CRO. No language or geographical restrictions were applied. Search strategies were tailored to each database and combined terms related to melanoma, teledermatology, telemedicine, mobile health, artificial intelligence, diagnosis, prognosis, triage, and access to care. The complete search strategies are available in Supplementary Materials S1.

### 2.4. Study Selection

**Figure 1:**
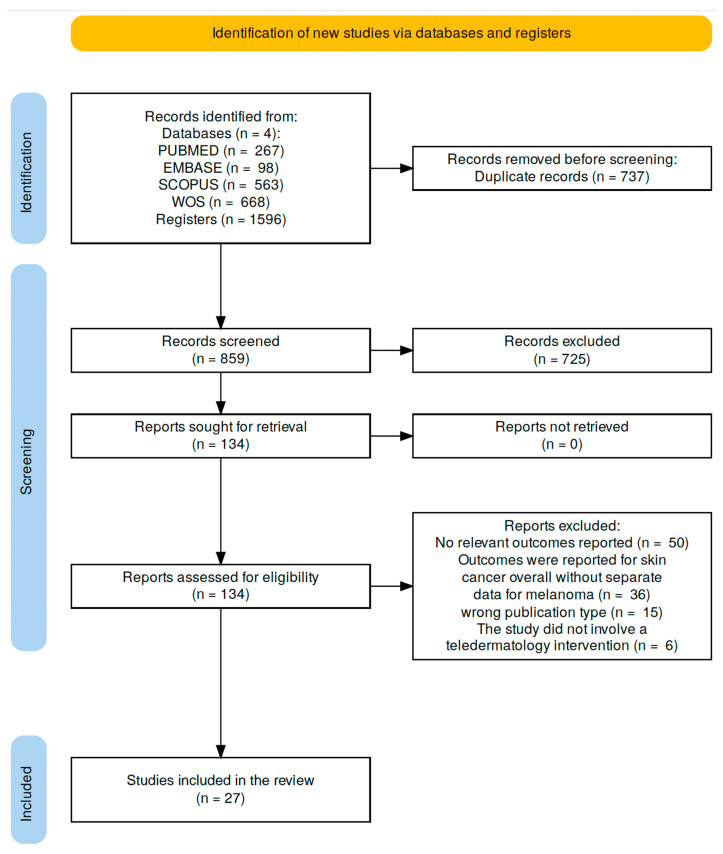
PRISMA 2020 Flow Diagram of Study Selection Process.

All retrieved references were imported into Rayyan for screening and de-duplication. Two reviewers (MLPR and MGV) independently screened titles and abstracts. In the case of disagreement during article selection, a third supervising researcher (MDSAR) made the final decision. A total of 1596 records were identified; 859 remained after removing duplicates. After screening, 134 full-text articles were reviewed, and 29 were ultimately included (Figure 1).

### 2.5. Data Extraction and Processing

Data were extracted by a multidisciplinary team. Extracted variables included study setting, population characteristics, type of TD intervention, comparator group, outcome measures, and main findings relevant to the review objectives. A qualitative synthesis was performed due to the heterogeneity in study designs, outcome measures, and reporting standards.

### 2.6. Risk of Bias Assessment

The risk of bias of the included studies was assessed using the Joanna Briggs Institute (JBI) Critical Appraisal Checklists [17], selecting the most appropriate tool based on each study’s design (diagnostic accuracy, analytical cross-sectional, cohort, or quasi-experimental). The overall risk was graded as low, moderate, or high according to the number and relevance of criteria fulfilled. Studies not compatible with standard JBI checklists—such as discrete choice experiments or descriptive surveys—were assessed narratively based on methodological transparency and relevance to the review objectives.

## 3. Results

A total of 27 primary studies met the eligibility criteria and were grouped into five thematic domains: (i) diagnostic accuracy (sensitivity/specificity); (ii) prognostic impact (Breslow thickness); (iii) waiting-time metrics; (iv) patient- and clinician-reported satisfaction; and (v) economic outcomes. Across all domains, most interventions were store-and-forward TD services delivered from primary care or community settings to hospital dermatologists; fewer studies assessed synchronous video TD or consumer-directed mobile apps.

### 3.1. Diagnostic Accuracy (Sensitivity and Specificity)

Ten studies reported diagnostic accuracy outcomes for melanoma triage (Table 1). Reported sensitivity for TD pathways ranged from 6.8% to 100%, and specificity from 30.4% to 99.5%, indicating substantial variability across studies [18,19,20,21,22,23,24,25,26,27].

Three studies evaluated the diagnostic accuracy of teledermatology using macroscopic images alone. Cazzaniga et al. (2019) [21] reported a sensitivity of 92.9% and a specificity of 80.3% for the triage of lesions submitted during a public screening campaign via a mobile application, showing promising performance despite the absence of dermoscopy. In the study by Wolf et al. (2013) [19], a teledermatologist assessing macroscopic images reached a sensitivity of 98.1% but at the expense of a very low specificity (30.4%), likely reflecting a cautious strategy that favoured over-referral in the absence of dermoscopic detail. In contrast, Jobbágy et al. (2022) [23] analysed real-world data from a primary care app-based triage pathway and reported lower primary-diagnosis sensitivity (66.7%) but high specificity (97.1%), with improved sensitivity (93.3%) when multiple differential diagnoses were considered. Overall, these macroscopic–image–only approaches demonstrated variable performance, with a general trend toward prioritising sensitivity over specificity—a conservative strategy likely adopted to minimise the risk of failing to detect melanoma, which could have serious clinical consequences.

Incorporating teledermoscopy generally improved diagnostic performance. Tan et al. (2010) [18], in a prospective dual-reader study, reported 100% sensitivity for one reader and 93.3% for another, with specificity ranging from 98.4% to 100%. Congalton et al. (2015) [20] observed a sensitivity of 96% and a specificity of 62% in a large cohort referred from primary care, while Fazil Jaber et al. (2023) [24] found slightly lower sensitivity (75%) but higher specificity (83.9%) when comparing TD with FTF consultations. Koop et al. (2023) [25], in a national teledermatoscopy programme, reported balanced metrics with sensitivity and specificity both above 90%. More recently, Gafoor et al. (2024) [26] demonstrated 100% sensitivity and 89% specificity using patient-acquired smartphone dermoscopy, and Zazo et al. (2024) [27] achieved a sensitivity of 98.9%, although specificity was not reported. These findings support the added value of dermoscopic imaging, even in patient-led scenarios, and highlight how improvements in smartphone-compatible dermatoscopes have enhanced the reliability of TD-based melanoma triage.

Two studies focused more specifically on artificial intelligence (AI)–assisted strategies. Wolf et al. (2013) [19] compared four smartphone applications against histologically confirmed melanoma diagnoses. Three of these apps operated as fully automated AI-based systems and demonstrated highly variable performance, with sensitivities ranging from 6.8% to 70% and specificities from 37.0% to 93.7%. These results reflect divergent design priorities: some apps maximised specificity by flagging only very high-risk lesions, whereas others prioritised sensitivity but generated excessive false positives. In comparison, the teledermatologist achieved higher sensitivity but low specificity, again suggesting a conservative approach in ambiguous cases. Jahn et al. (2022) [22] evaluated the SkinVision^®^ (SkinVision^®^ B.V., Amsterdam, The Netherlands, App-Version 6.8.1) AI-based risk stratification app in a cohort with an intentionally high proportion of melanoma cases. The standalone AI model achieved a sensitivity of 83% and a specificity of 60%. When dermatologists integrated the AI-generated risk scores into their assessment, specificity increased modestly to 88%, while sensitivity remained unchanged. Notably, the unaided dermatologist showed better overall diagnostic performance than both AI-supported and AI-alone strategies, achieving the same sensitivity (83%) but a higher specificity of 95%. These findings suggest that while current AI tools can replicate expert-level sensitivity, they may not enhance—and can even compromise—specificity in already experienced hands. Their clinical utility may be greater when assisting non-specialist clinicians, particularly in triage or primary care settings where diagnostic expertise is limited

### 3.2. Prognostic Impact (Breslow Thickness)

Seven studies reported on prognostic indicators, with Breslow thickness being the most consistently available outcome (Table 2). The impact of TD on tumour stage at diagnosis varied across settings [20,27,28,29,30,31,32]. All prognostic evidence came from observational studies, including one prospective multicentre study, one descriptive longitudinal study, four retrospective cohort or audit studies, and one retrospective cross-sectional analysis. No randomised controlled trials were identified. The reliance on observational evidence, with heterogeneous designs and populations, limits the strength of conclusions regarding the impact of TD on Breslow thickness.

Several studies suggested that TD was associated with earlier-stage melanoma diagnosis. In Spain, Ferrándiz et al. (2012) [28] observed a significantly lower mean Breslow thickness in patients referred via TD compared to FTF pathways (1.06 mm vs. 1.64 mm; *p* = 0.03). Moreover, the proportion of early-stage tumours (Tis + T1a) was higher in the TD group (70.1% vs. 56.9%; odds ratio 1.96, *p* = 0.04). In Sweden, Börve et al. (2015) [30] found a lower median Breslow thickness among TD-diagnosed melanomas compared to paper-based referrals (1.0 mm vs. 2.2 mm), and a greater proportion of in situ lesions (46% vs. 35%). Congalton et al. (2015) [20], using data from New Zealand’s Virtual Lesion Clinic (VLC), reported a median Breslow thickness of 0.69 mm among invasive melanomas detected through TD, with 62% measuring less than 1.0 mm and lacking ulceration or mitotic activity. Similarly, Teague et al. (2022) [31], in a larger retrospective audit from the same region, found a median Breslow thickness of 0.60 mm and a favourable in situ to invasive melanoma ratio of 1.31 in lesions triaged through TD, suggesting effective early detection.

In contrast, other studies associated TD with worse prognostic indicators. In the U.S. Veterans Affairs network, Karavan et al. (2013) [29] reported a significantly higher mean Breslow thickness in the TD group compared to FTF (1.91 mm vs. 1.14 mm; *p* = 0.03). Thick melanomas (>4 mm) were more frequent in the TD group (14% vs. 4%), and thin melanomas (<1 mm) were less common (59% vs. 74%). More recently, Jaklitsch et al. (2024) [32] documented similar trends, mean Breslow thickness was significantly higher in TD (0.95 mm) than in FTF referrals (0.40 mm; *p* < 0.001), and a significantly greater proportion of TD patients presented with invasive melanomas (74.3% vs. 46.6%), ulceration (32.0% vs. 9.0%), and histologically aggressive subtypes (22.9% vs. 7.9%). These findings suggest that, in some systems, TD may be selectively used for more complex or advanced lesions or suffer from delays that offset its potential advantages.

Finally, Zazo et al. (2024) [27], using real-world data from a Swedish county healthcare system, reported no significant differences in prognosis between TD and conventional pathways. The mean Breslow thickness of invasive melanomas in the TD group was 1.55 mm, with 60% of cases presenting with a thickness ≤ 1.0 mm. However, the comparison between TD and non-TD referrals did not reach statistical significance (*p* = 0.4), suggesting comparable diagnostic timing across both modalities in that setting.

Altogether, these results highlight that TD may enable earlier melanoma detection in structured systems with streamlined workflows and rapid access to biopsy or excision. However, its effectiveness is not universal and may depend on healthcare infrastructure, referral dynamics, and how TD is integrated into the broader care pathway.

### 3.3. Waiting-Time Outcomes

Several studies assessed the impact of TD on time to first dermatological assessment and/or definitive treatment (Table 3). Most of them reported significant reductions in waiting times when TD was used [20,25,30,32,33,34,35,36,37].

May et al. (2008) [33] found that patients whose referrals included digital photographs were seen in clinic much sooner than those without images: median waiting time to dermatology for melanoma was 14 days with TD, versus 24 days for urgent referrals, 44 days for “soon” priority, and up to 130 days for routine referrals. In the latter group, only three cases of melanoma had been classified as routine, while the vast majority of routine referrals corresponded to benign lesions. Time to treatment was also shorter in the TD group (median 21.5 vs. 41–136 days depending on priority).

In Sweden, Börve et al. (2015) [30] showed that TD referrals led to dermatologist review within 24 h (median 1.8 h) and surgical management within 9–12 days, compared to 14–17 and 35–62 days, respectively, for paper-based referrals (*p* < 0.0001 and *p* = 0.028). Similarly, Congalton et al. (2015) [20] reported a substantial reduction in waiting time to first assessment in New Zealand’s Virtual Lesion Clinic (9 vs. 26.5 days), although the time to excision remained variable (median 40 days, range 14–210). Dahlén Gyllencreutz et al. (2017) [34] observed that TD significantly improved prioritisation of invasive melanoma (98% vs. 62%; *p* = 0.012), allowing more patients to be booked directly for surgery (91% vs. 36%).

The largest time differentials were reported by Jaklitsch et al. [32] in 2024; the median time to first visit for melanoma was 0 days for TD versus 42 days for FTF, and time to biopsy was similarly reduced (6 vs. 47 days; *p* < 0.001). Koop et al. (2023) [25], analysing Estonia’s national TD programme, found a mean time to excision of 45.5 days and to histological confirmation of 67.4 days. These outcomes were reported exclusively within the TD pathway, with no conventional referral comparator available; the authors interpreted the observed intervals as indicative of efficient system-level coordination.

However, not all studies reported significant improvements. Bouton et al. (2024) [36], in a cluster-randomised trial in France, found no meaningful difference in consultation delay for lesions requiring excision between the TD and standard-referral groups (56.5 vs. 63.7 days; *p* = 0.53), possibly due to sample size limitations and heterogeneity in GP engagement. Similarly, Sahin et al. (2024) [37], in a retrospective cohort study from Sweden, reported no statistically significant difference in time to melanoma excision before and after TD implementation (56.7 vs. 49.7 days; *p* = 0.705), although both groups fell within acceptable timeframes according to the authors.

Overall, the evidence suggests that TD can substantially reduce time to assessment and management when integrated into structured systems with streamlined communication and defined referral protocols. Nonetheless, its impact is not uniform across settings and appears to depend on local infrastructure, triage efficiency, and how promptly TD referrals are converted into definitive treatment actions.

### 3.4. Satisfaction

Overall, satisfaction with TD was high across studies (Table 4), particularly when dermatologist involvement was maintained. Patients appreciated TD’s convenience, ease of use, and potential to reduce unnecessary procedures and delays [22,38,39,40,41,42,43,44].

In Australia, several studies demonstrated strong patient acceptance of mobile TD tools. Horsham et al. (2016, 2020) [39,44] evaluated patient-performed TD using smartphone applications and found that over 90% of participants considered the technology easy to use and were motivated to perform regular skin self-examinations. However, some users needed assistance with image capture or expressed anxiety while awaiting results. Spinks et al. (2015) [38] showed that patients strongly preferred TD with dermatologist review over traditional consultations, citing improved accuracy and reduced disruption to daily life. Similar findings were reported by Koh et al. (2018) [41], with 95% of users willing to submit images via mobile app, although some expressed concerns regarding data privacy and the reliability of the diagnosis.

High satisfaction was also reported in other regions. Chin et al. (2020) [42] noted >90% user satisfaction with an AI-based mobile app in Taiwan. In Georgia and Belgium, both patients and physicians valued TD for its perceived reliability, time savings, and ease of integration into care workflows [40,43].

Trust in artificial intelligence (AI) tools alone was more limited. In the Swiss study by Jahn et al. (2022) [22], patients expressed full trust in dermatologists and total-body imaging systems but showed lower confidence in the standalone AI app (SkinVision^®^), particularly among high-risk individuals. While most participants believed AI could support decision-making, none preferred the app alone as a screening method. Dermatologists also reported low confidence in its diagnostic utility.

These findings suggest that TD is well-received by patients and providers, especially when guided by dermatologists. By contrast, the acceptance of AI-based tools largely depends on their incorporation into workflows supervised by medical experts.

### 3.5. Economic Outcomes

Only two studies reported cost-related outcomes for TD in the management of melanoma (Table 5), both showing substantial savings compared to conventional care [20,40].

Congalton et al. (2015) [20], evaluating the Virtual Lesion Clinic (VLC) model in New Zealand, estimated a cost reduction of NZ$364,330 over the study period, equivalent to approximately NZ$1174 per patient seen. These savings were attributed to more efficient triage, reduced need for in-person consultations, and timely surgical intervention. While the analysis did not include a formal cost-effectiveness model, the scale of the reduction suggests strong economic potential for TD in structured referral pathways.

In Georgia, Kirtava et al. (2016) [40] reported that traditional FTF dermatology care was at least 3.65 times more expensive than TD in rural outpatient settings. Though specific cost components were not itemised, the study highlighted TD’s feasibility and affordability in low-resource environments with limited access to specialist care.

Despite the limited number of studies, current data suggest that TD can generate meaningful cost savings, particularly when integrated into systems with streamlined workflows and high patient volumes. Further economic evaluations—including direct and indirect costs—are needed to clarify its cost-effectiveness across diverse healthcare settings.

Flowchart illustrating the identification, screening, eligibility assessment, and inclusion of studies in the systematic review. A total of 1596 records were identified through database searches. After removing duplicates and screening titles and abstracts, 134 full-text reports were sought for retrieval. All of them were assessed for eligibility, and 27 studies were ultimately included in the qualitative synthesis. Reasons for exclusion are detailed in the diagram.

## 4. Discussion

This systematic review highlights the potential of TD to improve access, diagnostic accuracy, and efficiency in the management of cutaneous melanoma. However, the overall strength of the evidence is tempered by methodological heterogeneity and risk of bias across studies. Despite these limitations, the accumulated evidence points toward specific scenarios in which TD—particularly when supported by dermoscopy and expert interpretation—can enhance early detection and optimise care pathways.

Following this, the diagnostic performance of TD in melanoma was generally high, particularly in studies incorporating dermoscopic images and expert interpretation [18,20,24,25,26,27]. Reported sensitivities frequently exceeded 90%, indicating that TD is a reliable strategy for the initial triage of suspicious lesions. However, specificity varied markedly depending on the type of images used and the clinical setting. Studies based solely on macroscopic images tended to overtriage [19,21,23]. This is likely due to the limited visual detail available without dermoscopy. In contrast, the addition of dermoscopic images—when properly captured—improved both sensitivity and specificity [18,20,24,25,26,27]. Yet in real-world practice, up to 36% of dermoscopic images may be of insufficient quality due to suboptimal lighting, resolution, or focus. These limitations highlight the importance of standardised acquisition protocols and adequate training to ensure reliable interpretation and maintain diagnostic accuracy [45].

AI tools have also been explored as diagnostic aids in TD, but their performance remains inconsistent. Most models show acceptable sensitivity but often at the expense of lower specificity, and they are rarely trusted as standalone tools by dermatologists [19,22]. Wolf et al. reported that three out of four apps misclassified at least 30% of melanomas as low-risk or benign. The authors cautioned that relying on such tools in place of medical consultation—especially in the absence of regulatory oversight—may result in delayed diagnosis and potential harm to users [19]. Recent concerns raised by the EADV AI Task Force include a lack of external validation, limited representation of skin types, and the widespread availability of uncertified apps without clinical oversight. While AI may support non-specialist users in initial triage, its role in expert-guided TD remains limited [46]. Several studies have also demonstrated poor diagnostic accuracy for melanoma detection when compared to dermatologists [47,48,49]. Ensuring transparency, regulatory standards, and real-world testing is essential before broader clinical implementation [46].

Finally, recent evidence suggests that TD may miss clinically relevant incidental findings, particularly lesions not included in the photographic referral. For instance, Tejasvi et al. reported that 75% of melanomas diagnosed following a TD consult were not among the lesions initially submitted, but rather identified during subsequent in-person examinations. These findings underscore one of the main limitations of TD: the inability to perform a full-body skin examination, which may result in underdetection of clinically silent melanomas [50]. This concern was further illustrated in a recent randomised trial by Soyer et al. (2025) [51], in which 3D total-body photography with sequential dermoscopy review resulted in a higher number of excisions (905 vs. 622) but paradoxically fewer melanomas diagnosed compared with usual care (24 vs. 43). The authors suggested that reliance on image-based review without direct physical examination may contribute to both over-biopsy of benign lesions and underdetection of melanoma, emphasising the need to integrate TD with in-person assessments in high-risk patients. Moreover, TD also limits opportunities for patient education. To mitigate these risks, TD services should ensure timely in-person follow-up for high-risk individuals and incorporate structured patient education to promote regular skin self-examination. In addition, requesting panoramic body photographs alongside images of suspicious lesion(s) could provide a broader screening perspective and help detect incidental melanomas that might otherwise be overlooked. In selected primary care settings, sequential digital dermoscopy or even 3D total-body imaging could be used to deliver images for expert evaluation, although the feasibility of these approaches will inevitably vary depending on healthcare resources and infrastructure.

With respect to prognosis, data relied entirely on observational designs, including retrospective cohorts, cross-sectional analyses, audits, and one prospective observational study. This heterogeneity, along with the absence of RCTs, limits the strength of conclusions regarding the prognostic impact of TD. Several studies reported that TD was associated with earlier melanoma diagnosis, reflected in thinner Breslow thickness and a higher proportion of in situ tumours [20,28,30]. These results suggest that, when embedded in efficient referral systems, TD may facilitate faster identification and treatment of melanoma. However, this positive trend was not consistent across all settings. In particular, some U.S.-based cohorts reported significantly thicker melanomas in patients referred via TD compared to those diagnosed through conventional FTF pathways [29,32]. Such discrepancies likely reflect differences in healthcare system organisation, referral pathways, and the timeliness of transition from TD assessment to biopsy or excision. In some contexts, TD was even preferentially used for more complex or advanced lesions, which may have biased outcomes toward worse prognosis. Conversely, in systems with streamlined workflows and rapid access to surgical management, TD facilitated earlier diagnosis and thinner tumours. These findings underscore the importance of ensuring not only rapid TD assessment but also seamless integration with downstream services. Without timely access to biopsy or excision, the potential diagnostic benefits of TD may be diminished or even reversed [13]. In addition, patients living in rural or remote areas often face longer travel distances and higher costs associated with in-person assessment, which may discourage timely presentation. Socioeconomic constraints can further exacerbate these delays, leading to diagnosis at a more advanced stage. In such contexts, tumour thickness may reflect patient-related barriers rather than limitations inherent to the TD pathway. However, the extent to which distance, cost, or healthcare organisation individually contribute to these differences is not yet clear, highlighting an important evidence gap that warrants further investigation.

Regarding waiting times, most studies demonstrated that TD significantly reduced time to first dermatological evaluation and, in many cases, time to biopsy or surgical treatment. Median time to first visit was frequently shortened by several weeks in TD pathways compared to standard referrals. This improvement was particularly marked in health systems with well-integrated TD workflows, rapid dermatologist response, and prioritisation protocols [20,30,32,33]. However, a subset of studies reported no significant improvement in waiting times [36,37], suggesting that the impact of TD is highly dependent on broader organisational factors. Where infrastructure, coordination, or follow-up mechanisms are lacking, the introduction of TD alone may not be sufficient to expedite care. These findings highlight that TD is most effective when implemented as part of a streamlined, well-integrated clinical pathway rather than as an isolated intervention.

Patient satisfaction with TD was generally high across studies, with users valuing its convenience, accessibility, and time-saving potential. Acceptance was particularly strong when dermatologists were involved in the process, either directly or through asynchronous reviews [38,39,40,41,42,43,44]. In contrast, confidence in standalone AI-based mobile apps remained low, especially among high-risk patients and specialists, suggesting that current AI tools are better positioned as adjuncts rather than independent diagnostic solutions [22].

Finally, the available data suggest that TD may offer meaningful cost advantages compared to conventional FTF care—particularly in health systems with well-structured referral pathways or in resource-limited settings. Reported savings were primarily attributed to a reduction in unnecessary in-person visits, more efficient triage, and earlier initiation of treatment, all of which contribute to optimised use of clinical resources [20,40]. However, only two studies specifically addressed costs, and they were conducted in very different healthcare contexts, which limits comparability. While both suggested potential savings, the small evidence base and lack of comprehensive cost-effectiveness modelling preclude broad generalization.

Smak Gregoor et al. (2023) [52] evaluated an AI-based mobile application for skin cancer detection in a population-based cohort of over 285,000 participants, reporting that its use could reduce unnecessary dermatology visits and potentially lower healthcare expenditures at scale. By contrast, Lindsay et al. (2025) [53], in a randomised trial including 314 high-risk patients, found that 3D total-body photography combined with sequential dermoscopy and TD review was not cost-effective compared with usual care. The intervention increased per-patient costs by approximately USD 4200 over two years, without significant differences in melanoma incidence or stage at diagnosis.

These contrasting findings highlight the heterogeneity of economic outcomes depending on the technology evaluated and the health system context. As a result, while the economic potential of TD appears promising, current evidence remains insufficient to support generalisable conclusions. Future studies should incorporate standardised economic endpoints and compare TD against existing care pathways from both healthcare system and societal perspectives.

### 4.1. Clinical Implications and Future Directions

The findings of this review support the clinical integration of TD as a valuable strategy to enhance melanoma detection, particularly in systems with limited dermatological access or long referral delays. TD, when supported by dermoscopy and expert interpretation, may achieve diagnostic accuracy comparable to FTF evaluation while improving efficiency and patient satisfaction. Its application could be especially impactful in primary care or underserved regions where dermatology expertise is scarce. However, careful system design is essential to avoid unintended diagnostic delays or increased burden on referral pathways. At the same time, diagnostic variability between TD assessors and across studies remains a recognised limitation [54], potentially reducing consistency of outcomes. Artificial intelligence offers a promising approach to improving diagnostic consistency by providing more standardised support. At present, AI appears most suited to assisting non-expert clinicians in triage and lesion recognition rather than replacing specialist assessment. Future research should define optimal strategies for integrating AI into referral pathways and evaluate its safety, accuracy, and acceptability across diverse clinical contexts.

There is also a need for robust implementation studies and prospective cohort designs with long-term follow-up to determine whether TD improves not only diagnostic timing but also melanoma-specific outcomes. Standardised outcome reporting and economic evaluations—including cost-effectiveness and budget impact analyses—would further strengthen the evidence needed to inform healthcare planning and decision-making.

### 4.2. Limitations of the Review

This review has several limitations. First, the included studies varied widely in design, population, and outcome reporting, which limited the possibility of conducting a quantitative meta-analysis and necessitated a descriptive synthesis. Although the JBI Critical Appraisal Checklists [17] were systematically applied to all eligible studies, certain designs (such as discrete choice experiments or descriptive surveys) were not compatible with these tools and were therefore assessed narratively. To ensure transparency, all completed checklists are provided in Supplementary Materials S2. Second, multiple studies presented a moderate to high risk of bias, with potential issues such as self-selected samples, verification bias, incomplete follow-up, and absence of histological confirmation in all lesions. Third, publication bias cannot be ruled out, particularly the underrepresentation of negative or neutral findings. Finally, as teledermatology technologies are evolving rapidly, some older studies may not reflect current practice, while newer AI- or app-based interventions remain insufficiently validated.

## 5. Conclusions

TD has emerged as a promising strategy to improve the diagnosis and management of cutaneous melanoma, particularly in healthcare systems facing rising demand and limited specialist capacity. This systematic review highlights its potential to enhance diagnostic accuracy—especially when dermoscopy and expert interpretation are incorporated—while reducing waiting times and improving patient satisfaction. Although evidence also points to possible prognostic and economic benefits, these results are not consistent across all settings and appear to be influenced by how TD is implemented and integrated into existing care pathways. The role of AI remains controversial and should be approached with caution, particularly in direct-to-consumer applications. Further high-quality studies are needed to assess the long-term clinical impact, cost-effectiveness, and optimal implementation strategies for TD in real-world melanoma care.

## Figures and Tables

**Table 1:** Diagnostic Accuracy of Teledermatology for Melanoma (Sensitivity and Specificity).

Author & Year	Country/Setting	Study Design	Population	TD Intervention	Comparator	Melanoma Results	Risk of Bias
Tan et al., 2010 [18]	New Zealand/Waikato Hospital lesion clinic	Prospective diagnostic accuracy study	200 patients; 491 lesions; 2 dermatologists	Store-and-forward TD with macroscopic + dermoscopic images reviewed remotely	FTF consultation and histopathology	Sensitivity for melanoma: Derm A: 100% (vs. FTF & histology) Derm B: 93.3% (vs. FTF), 100% (vs. histology) Specificity for melanoma: Derm A: 98.7% (vs. FTF), 98.4% (vs. histology) Derm B: 100% (vs. FTF), 99.5% (vs. histology)	Risk of bias discussed; measures to minimise recall and selection bias described, although no formal tool used.
Wolf et al., 2013 [19]	USA/University dermatology department	Case-control diagnostic accuracy study	188 digital images (60 melanoma, 128 benign); histopathologically confirmed	Four smartphone apps: mobile app 1: AI analysis with user-confirmed lesion border mobile app 2: Fully automated AI (“melanoma” vs. “looks good”) mobile app 3: AI with risk score (low/medium/high); “medium” counted as positive mobile app 4: Store-and-forward TD image to a dermatologist	Histopathology	Sensitivity/Specificity: mobile app 1: 70.0%/39.3% mobile app 2: 69.0%/37.0% mobile app 3: 6.8%/93.7% mobile app 4: 98.1%/30.4%. mobile app 4 (teledermatologist) was significantly more sensitive than AI apps (*p* < 0.001 vs. mobile app 1 & 3; *p* = 0.02 vs. mobile app 2).	Not formally assessed; descriptive study without discussion of potential bias or methodological limitations.
Congalton et al., 2015 [20]	New Zealand/Waitematā District Health Board	Retrospective observational study	310 patients; 613 lesions assessed via VLC; 129 lesions excised; 48 melanomas confirmed (23 in situ, 24 invasive, 1 metastatic)	Store-and-forward TD with macroscopic + dermoscopic images evaluated remotely	FTF consultation and histopathology	Sensitivity of 96% (95% CI: 86–99%) Specificity of 62% (95% CI: 50–73%)	Not formally assessed; no discussion of potential sources of bias or methodological limitations.
Cazzaniga et al., 2019 [21]	Italy/General population in Bergamo province	Prospective validity study within public awareness campaign	232 adults who submitted lesion photos via mobile app; followed by clinical exam	Mobile app allowing users to send images of suspicious lesions to dermatologists for triage	FTF whole-body clinical examination	sensitivity was 92.9% (95% CI 66.1–99.8%), specificity 80.3% (95% CI 74.4–85.3)	Not formally assessed; self-selection bias likely due to participant profile and lack of data on non-users.
Jahn et al., 2022 [22]	Switzerland/University Hospital of Basel	Prospective, single-centre, observational cohort	114 patients (55 high-risk, 59 with melanoma); 1204 lesions; 61 lesions with histology available	mobile app SkinVision^®^ (macroscopic image-based AI risk stratification)	Dermatologists; 2D FotoFinder ATBM^®^; 3D Vectra; histopathology	Sensitivity/Specificity: SkinVision^®^ 83%/60.0%; 2D FotoFinder ATBM^®^ 83%/40.0%; 3D Vectra 83%/63.6%. Dermatologists 83%/92.7% Dermatologists + AI scores 83%/88%	Not formally assessed; single-centre design acknowledged as limitation.
Jobbágy et al., 2022 [23]	Hungary/Semmelweis University, Budapest	Retrospective single-centre case-control study	Subset of 100 lesions (30 malignant, 70 benign) retrospectively selected from a larger dataset of 779 cases (during first COVID-19 wave)	Store-and-forward TD using mobile app (photos and questionnaire submitted)	FTF consultation and histopathology	Sensitivity (melanoma): Primary diagnosis: 66.7% (95% CI: 41.7–84.8) Aggregated diagnosis (including any differencial diagnosis during teledermatology): 93.3% (95% CI: 70.2–99.7) Specificity (melanoma): Primary: 97.1% (95% CI: 95.7–98.1) Aggregated: 96.3% (95% CI: 94.8–97.5)	Not formally assessed; retrospective design with acknowledged limitations including lack of dermoscopy and variable image quality.
Fazil Jaber et al., 2023 [24]	Sweden/Ryhov County Hospital, Jönköping	Cross-sectional, retrospective study	112 TD patients and 134 FTF patients with suspected melanoma or atypical melanocytic lesions	Store-and-forward TD with clinical + dermoscopic images captured by GPs and reviewed by dermatologists	FTF consultation and histopathology	TD showed 75% sensitivity and 83.9% specificity vs. 47.8% and 81.2% for FTF	Not formally assessed; retrospective design, high-quality images only, real-world setting
Koop et al., 2023 [25]	Estonia/National primary care network	Retrospective database study	4748 teledermatoscopy cases from 3403 patients, 50 melanomas (October 2017–August 2019)	Store-and-forward TD mobile teledermatoscopy (smartphone + dermatoscope, dermatologist-reviewed)	Histopathology	Sensitivity: 90.5% (95% CI: 69.6–98.8); Specificity: 92.6% (95% CI: 91.8–93.3).	Formally assessed using STARD and ISPOR standards; limitations of retrospective design discussed.
Gafoor et al., 2024 [26]	UK/Dermatology clinics (2 sites)	Service evaluation (prospective)	78 patients (390 image assessments)	Patient-led Mobile TD with Dyplens™ dermoscope and smartphone; images reviewed by 5 dermatologists	FTF consultation and histopathology	Sensitivity: 100% for melanoma Specificity: 89% for melanoma	Not formally assessed.
Zazo et al., 2024 [27]	Sweden/Västerbotten County healthcare system	Retrospective cross-sectional study	135 patients diagnosed with melanoma, 95 via TD	Store-and-forward TD (macro + dermoscopic images) from primary care to dermatology	FTF consultation and histopathology	TD showed sensitivity of 98.9%.	Not formally assessed; retrospective design with good coverage and real-life data

Summary of studies assessing the diagnostic performance of teledermatology compared to face-to-face consultation and/or histopathology, including the type of images used, the presence of dermoscopy, and AI support when applicable. Abbreviations: TD = teledermatology; FTF = face-to-face; AI = artificial intelligence; GP = general practitioner.

**Table 2:** Prognostic Indicators in Patients Diagnosed via Teledermatology vs. Face-to-Face Referral.

Author & Year	Country/Setting	Study Design	Population	TD Intervention	Comparator	Melanoma Results	Risk of Bias
Ferrándiz et al., 2012 [28]	Spain/Hospital Universitario Virgen Macarena, Seville	Descriptive and longitudinal observational study	201 patients with primary cutaneous melanoma	Store-and-forward TD TD (TD)system using standard digital photographs transmitted via intranet or email	Conventional FTF referral system	Mean Breslow thickness significantly lower in TD group (1.06 mm vs. 1.64 mm, *p* = 0.03); higher rate of early-stage tumours (Tis + T1a: 70.1% vs. 56.9%, OR = 1.96, *p* = 0.04)	Not formally assessed; potential selection bias and loss to follow-up acknowledged as limitations.
Karavan et al., 2013 [29]	USA/Veterans Affairs network (Pacific NW)	Retrospective cohort study	529 Veterans with 567 pathology-confirmed melanomas (112 TD, 455 non-TD)	Store-and-forward TD TD with digital photography by trained technicians	Conventional FTF referral system	Mean Breslow: 1.91 mm (TD) vs. 1.14 mm (non-TD) (*p* = 0.03). Higher % of thick melanomas (>4 mm) in TD group (14% vs. 4%, *p* < 0.01). Lower proportion of thin melanomas (<1 mm) in TD (59% vs. 74%, *p* = 0.06). In situ melanoma: 44% (TD) vs. 42% (non-TD).	Not formally assessed; descriptive study without discussion of potential bias or methodological limitations.
Börve et al., 2015 [30]	Sweden/20 PHCs and 2 hospitals	Open, multicentre, prospective observational study	772 patients with skin lesions of concern referred via smartphone TDS	Smartphone TD using iDoc24 PRO mobile app and dermoscope	Conventional FTF referral system	TDS: 46% of MM were in situ vs. 35% with paper. Median Breslow: 1.0 mm (TDS) vs. 2.2 mm (paper).	Not formally assessed; controlled design with robust sample.
Congalton et al., 2015 [20]	New Zealand/Waitematā District Health Board	Retrospective observational study	310 patients; 613 lesions assessed via VLC; 129 lesions excised; 48 melanomas confirmed (23 in situ, 24 invasive, 1 metastatic)	Store-and-forward TD TD with macroscopic + dermoscopic images evaluated remotely	Conventional FTF referral system	The median Breslow thickness of the 24 invasive primary lesions was 0.69 mm (range 0.3–5.0 mm). Sixty-two percent were <1.0 mm in thickness without ulceration or mitoses.	Not formally assessed; small sample size, good clinical relevance
Teague et al., 2022 [31]	New Zealand/Waitematā District Health Board	Retrospective audit (5 years)	810 patients; 3546 lesions referred to VLC; 504 excised lesions analysed	VLC: TD triage of pigmented lesions via MoleMap imaging and remote dermatologist review	None (descriptive retrospective audit)	The mean Breslow thickness for invasive melanoma was 1.26 mm (95% CI: 0.90–1.61, median 0.60 mm), and the IM:MIS ratio was 0.76 (80 invasive melanoma: 105 melanoma in situ).	Not formally assessed; acknowledged limitations include incomplete follow-up of excision recommendations and potential selection bias.
Jaklitsch et al., 2024 [32]	USA/University of Pittsburgh Medical Center	Retrospective cohort (2020–2022)	836 melanomas (35 TD, 801 FTF)	Asynchronous (eDerm) and synchronous video TD for triage	Conventional FTF referral system	TD vs. FTF: Median Breslow: 0.95 vs. 0.40 mm (*p* < 0.001) Invasive melanomas: 74.3% vs. 46.6% (*p* = 0.001) Ulcerated: 32.0% vs. 9.0% (*p* = 0.002) Aggressive subtype: 22.9% vs. 7.9% (*p* = 0.007)	Not formally assessed; retrospective design and single-centre setting may introduce selection bias and limit generalisability.
Zazo et al., 2024 [27]	Sweden/Västerbotten County healthcare system	Retrospective cross-sectional study	135 patients diagnosed with melanoma, 95 via TD	Store-and-forward TD (macro + dermoscopic images) from primary care to dermatology	Conventional FTF referral system	The mean Breslow thicknes of invasive melanomas was 1.55 mm and the proportion of patients with Breslow thickness ≤ 1.0 mm was 60%. There were no significant differences in Breslow thickness (*p* = 0.4), between patients evaluated with or without TD consultations.	Not formally assessed; retrospective design with good coverage and real-life data

Breslow thickness and stage distribution of melanomas diagnosed through teledermatology compared with conventional referral pathways. Abbreviations: TD = teledermatology; FTF = face-to-face; VLC = Virtual Lesion Clinic; MM = malignant melanoma; MIS = melanoma in situ.

**Table 3:** Impact of Teledermatology on Waiting Times for Diagnosis and Treatment.

Author & Year	Country/Setting	Study Design	Population	TD Intervention	Comparator	Melanoma Results	Risk of Bias
May et al., 2008 [33]	UK/Lanarkshire dermatology clinics	Prospective observational comparative study	76 patients with melanoma and SCC	Store-and-forward TD TD with digital images and dermoscopy reviewed for triage	Conventional referral without images	Median time to clinic (melanoma): With photos: 14 days Without photos: 24 days (urgent), 44 days (‘soon’), 130 days (routine) Median time to treatment (melanoma): With photos: median 21.5 days Without photos: 41 days (urgent), 51 days (‘soon’), 136 days (routine). No routine-priority patients without photos were treated within 62 days; all but one with photos were.	Not formally assessed; observational design without discussion of potential sources of bias.
Börve et al., 2015 [30]	Sweden/20 PHCs and 2 hospitals	Open, multicentre, prospective observational study	772 patients with skin lesions of concern referred via smartphone TDS	Smartphone TD using iDoc24 PRO mobile app and dermoscope	Conventional referral without images	TD: first visit in 9/10 days and surgery in 9/12 days for MM/MMIS vs. 14/17 and 35/62 days with paper (*p* < 0.0001 and *p* = 0.028). Dermatologist response in <24 h (median 1.8 h; TD) vs. 4-day delay (paper).	Not formally assessed; controlled design with robust sample.
Congalton et al., 2015 [20]	New Zealand/Waitematā District Health Board	Retrospective observational study	310 patients; 613 lesions assessed via VLC; 129 lesions excised; 48 melanomas confirmed (23 in situ, 24 invasive, 1 metastatic)	Store-and-forward TD TD with macroscopic + dermoscopic images evaluated remotely	Conventional referral without images	Reduced wait time to first assessment (9 vs. 26.5 days). The median wait time for diagnostic excision from VLC assessment report was 40 days for lesions suspicious of melanoma (range 14–210 days)	Not formally assessed; no discussion of potential sources of bias or methodological limitations.
Dahlén Gyllencreutz et al., 2017 [34]	Sweden/Primary care to dermatology services	Comparative study (TDS vs. paper-based referrals)	157 cases (80 TDS, 77 paper); evaluated by 6 dermatologists	Mobile TD: macroscopic + dermoscopic images assessed remotely	Conventional referral without images	TDS significantly improved correct prioritisation of invasive melanoma (98% vs. 62%; *p* = 0.012) and allowed more patients to be booked directly for surgery (91% vs. 36%)	Not formally assessed; methodological limitations discussed, including interobserver variability and potential misclassification in triage decisions.
Teoh & Oakley, 2022. [35]	New Zealand/Te Whatu Ora Waikato	Retrospective service review (9 years, 5 months)	6479 patients; 11,005 lesions imaged; 330 histologically confirmed melanomas	Nurse-led imaging clinics using digital and dermoscopic photography for remote diagnosis by dermatologists	None (descriptive study)	median waiting time of 44.5 (mean 57.9; range 8–218) days for imaging and a median waiting time of 63 (mean 63.2; range 28–94) for the first treatment received	Not formally assessed; retrospective design with limited detail on comparator groups or bias control methods.
Koop et al., 2023 [25]	Estonia/National primary care network	Retrospective database study	4748 teledermatoscopy cases from 3403 patients (October 2017–August 2019)	Store-and-forward TD mobile teledermatoscopy (smartphone + dermatoscope, dermatologist-reviewed)	None	Mean time to excision: 45.5 days; to histology: 67.4 days.	Formally assessed using STARD and ISPOR standards; limitations of retrospective design discussed.
Bouton et al., 2024 [36]	France/Primary care practices (Nantes region)	Cluster-randomised controlled trial	250 patients referred for suspected melanoma (125 per group)	Email transmission of smartphone photos by GPs to dermatologists	Conventional referral without images	No significant difference in consultation delay for lesions requiring resection (56.5 vs. 63.7 days, *p* = 0.53).	Not formally assessed; strengths include randomised design, but limitations in statistical power and lack of standardised bias assessment tool.
Jaklitsch et al., 2024 [32]	USA/University of Pittsburgh Medical Center	Retrospective cohort (2020–2022)	836 melanomas (35 TD, 801 FTF)	Asynchronous (eDerm) and synchronous video TD for triage	Conventional referral without images	TD vs. FTF: Median time to first visit: 0 vs. 42 days (*p* < 0.001) Median time to biopsy: 6 vs. 47 days (*p* < 0.001)	Not formally assessed; retrospective design and single-centre setting may introduce selection bias and limit generalisability.
Sahin et al., 2024 [37]	Sweden/Östergötland County, 3 PHCs	Retrospective cohort study (Pre vs. Post implementation)	2137 patients with skin tumours, including 44 melanomas	Store-and-forward TD TD with macroscopic and dermoscopic images	Conventional referral without images	No significant difference in melanoma management times (PreT: 56.7 vs. PostT: 49.7 days until excision of a melanoma; *p* = 0.705)).	Not formally assessed; naturalistic design, representative sample

Comparison of delays to first dermatological evaluation, biopsy, or excision between teledermatology and traditional referral systems. Abbreviations: TD = teledermatology; FTF = face-to-face; PHC = primary healthcare centre; VLC = Virtual Lesion Clinic.

**Table 4:** Patient and Clinician Satisfaction with Teledermatology in Melanoma Care.

Author & Year	Country/Setting	Study Design	Population	TD Intervention	Comparator	Melanoma/PL Results	Risk of Bias
Spinks et al., 2015 [38]	Australia/Queensland	Discrete choice experiment with prior TD users	35 participants aged 50–64 at moderate/high melanoma risk	Store-and-forward TD Mobile TD with dermatologist image review	Skin self-exam, GP or skin clinic visit	Participants strongly preferred TD with dermatologist review. Key drivers: higher detection rate, fewer unnecessary removals, shorter time away from activities.	Not formally assessed; small sample and selection bias acknowledged as study limitations.
Horsham et al., 2016 [39]	Australia/Brisbane, community	Mixed-methods study: survey + home use trial	228 survey participants (mean age 57); 49 home users (mean age 59)	Patient-performed Mobile TD using dermatoscope + iPhone + Handyscope mobile app; Store-and-forward TD to dermatologist	None (descriptive study)	94% found the device easy to use; 86% said it motivated regular skin self-exam; 78% would use it again. However, 35% needed help submitting photos, 18% couldn’t image hard-to-see areas, and 12% felt anxious. 84% found the AC rule helpful. Trust in telediagnosis was moderate (46–56% agreement). Overall satisfaction was high, but confidence varied.	Not formally assessed; self-reported outcomes, participant self-selection and lack of clinical outcome verification may introduce bias.
Kirtava et al., 2016 [40]	Georgia/Outpatient dermatology clinics in Tbilisi and Adjara	Prospective observational study	584 patients screened for skin cancer, mainly pigmented lesions: 13 melanomas or Spitz tumours	Store-and-forward TD Mobile TD using DermLite DL3/DL1 attached to smartphones, with e-registry and remote review	Clinical diagnosis and follow-up; histopathology confirmation.	Approximately 60% of patients agreed or strongly agreed that TD is beneficial, reliable, and time- and cost-saving. Moreover, 90% of physicians agreed or strongly agreed that TD is beneficial and reliable, and 70% reported it saves time and money.	Not formally assessed; technical and operational limitations discussed, but no mention of methodological sources of bias.
Koh et al., 2019 [41]	Australia/Community (Brisbane)	Mixed methods: online survey + focus groups	88 online participants (mean age 38); 28 focus group participants (mean age 46)	Consumer-directed Mobile TD mobile app for photographing skin lesions and sending to a medical practitioner	None (descriptive study)	95% would use the mobile app to send images. Main perceived benefits: convenience (73%), ease of use (32%), cost reduction (13%). Barriers: privacy (25%), confidence in identifying lesions, system overload, and anxiety while awaiting results. Overall high acceptance.	Not formally assessed; limitations include single-centre design and potential diagnostic verification bias.
Chin et al., 2020 [42]	Taiwan/General public using mobile app	Cross-sectional satisfaction survey	1231 users of MoleMe mobile app for pigmented lesion evaluation	AI-based mobile app (MoleMe) analysing mole photos + clinical info; outputs risk and referral recommendation	None (descriptive study)	Over 90% satisfied (score 4–5); >75% strongly satisfied (score 5). High satisfaction in usability (95%), interaction (94%), impact on daily life (92%), and overall performance (94%). No significant differences by gender, age, or risk category.	Not formally assessed; self-reported, no diagnostic validation, limited to Asian population
Damsin et al., 2020 [43]	Belgium/PHCs near Liège	Pilot observational study (6 months)	80 patients, 105 lesions; PHCs with GP/nurse staff	Smartphone-based TD (TELESPOT) with macroscopic and dermoscopic imaging; remote review by dermatologists	None (descriptive study)	Patient satisfaction: 8.8/10 Comfort: 9.4 Tech confidence: 8.6 Trust in advice: 8.2 Physician satisfaction: 9.4/10 Integration: 8.6 Time efficiency: 9.4 Report usefulness: 9.6 Diagnostic support: 8.8 Value to PHC: 9.2 Skill improvement: 6.8	Not formally assessed; observational design and lack of control group may introduce selection and performance bias.
Horsham et al., 2020 [44]	Australia/Community-based, Brisbane	Randomised controlled trial	98 adults at high risk of skin cancer, aged 19–73, performing self-exams with mobile dermatoscope	Home-based Mobile TD using smartphone + dermatoscope for self-selected lesion imaging	Baseline self-report; no traditional FTF comparator	No change in melanoma worry; high baseline acceptance, but slight decline post-intervention; 92% found device easy to use, 71% would use again	Not formally assessed; self-reported outcomes, participant self-selection and lack of clinical outcome verification may introduce bias.
Jahn et al., 2022 [22]	Switzerland/University Hospital of Basel	Prospective, single-centre, observational cohort	114 patients (55 high-risk, 59 with melanoma); 1204 lesions; 61 lesions with histology available	mobile app SkinVision^®^ (macroscopic image-based AI risk stratification)	Dermatologists; 2D FotoFinder ATBM^®^; 3D Vectra; histopathology	Most patients expressed full trust in dermatologists (100%) and high confidence in 2D/3D total body imaging devices (>88%). In contrast, trust in the mobile app was notably lower (36% in high-risk patients, 49% in melanoma patients). While dermatologist assessments significantly reduced anxiety (81–89%), the mobile app alone achieved this in only about one-third of cases. The preferred screening method was dermatologist combined with 3D imaging (chosen by 51–64%), whereas no patient preferred the mobile app alone. Nearly all participants (95–98%) believed AI could support clinical decision-making. Dermatologists, however, reported low confidence in the mobile app (8.8%).	Not formally assessed; single-centre design acknowledged as limitation.

Overview of satisfaction outcomes reported by patients and healthcare professionals using teledermatology tools, including mobile applications, dermoscopy, and AI components. Abbreviations: TD = teledermatology; FTF = face-to-face; AI = artificial intelligence; GP = general practitioner; PHC = primary healthcare centre.

**Table 5:** Economic Outcomes Associated with Teledermatology Use in Melanoma Management.

Author & Year	Country/Setting	Study Design	Population	TD Intervention	Comparator	Melanoma Results	Risk of Bias
Congalton et al., 2015 [20]	New Zealand/Waitematā District Health Board	Retrospective observational study	310 patients; 613 lesions assessed via VLC; 129 lesions excised; 48 melanomas confirmed (23 in situ, 24 invasive, 1 metastatic)	Store-and-forward TD TD with macroscopic + dermoscopic images evaluated remotely	Conventional outpatient referral system	The VLC triage service resulted in an estimated cost reduction in NZ$364 330, or NZ$1174/patient seen (exchange rate Dec 2014: NZ$1.00 = US$ 0.77, Euro 0.62, UK£ 0.49).	Not formally assessed; no discussion of potential sources of bias or methodological limitations.
Kirtava et al., 2016 [40]	Georgia/Outpatient dermatology clinics in Tbilisi and Adjara (rural region)	Prospective observational study	584 patients screened for skin cancer, mainly pigmented lesions: 13 melanomas or Spitz tumours	Store-and-forward TD Mobile TD using DermLite DL3/DL1 attached to smartphones, with e-registry and remote review	Clinical diagnosis and follow-up; histopathology confirmation.	Traditional FTF care in the study region was at least 3.65 times more expensive than TD.	Not formally assessed; technical and operational limitations discussed, but no mention of methodological sources of bias.

Reported cost estimates and savings attributed to teledermatology implementation versus traditional models of care. Abbreviations: TD = teledermatology; FTF = face-to-face; VLC = Virtual Lesion Clinic.

## Data Availability

No new data were created or analyzed in this study. Data sharing is not applicable to this article.

## Supplementary Materials

The following supporting information can be downloaded at: https://www.mdpi.com/article/10.3390/cancers17172836/s1, Supplementary Materials S1: Search strategy; Supplementary Materials S2: Risk of Bias Assessments for Each Included Study.
