# Supplementary material for: Teledermatology vs. Face-to-Face Dermatology for the Diagnosis of Melanoma: A Systematic Review
Source: Cancers (Basel). 2025 Aug 29;17(17):2836. doi: 10.3390/cancers17172836 (PMC12427390; doi:10.3390/cancers17172836)
Supplement: Supplementary file 1 [file cancers-17-02836-s001.zip › cancers-3806241-supplementary.pdf]

Supplementary Material S1: Search strategy

1.PUBMED
Date: 20/12/2024

1.1.Search Strategy:

((((((((diagnosis[Text Word]) OR(diagnoses[Title/Abstract]) OR (prognosis[Text Word])) OR (prognoses[Title/Abstract])) OR (Breslow[Text Word])) OR ("Factor Prognostic" [tiab:~3])) OR ("Prognostic Factors" [tiab:~3])) OR (triage*[Title/Abstract])) OR (access[Title/Abstract])) AND (((((((((((("Telemedicine"[Mesh]) OR (Telemedicine[Text Word])) OR (mHealth[Title/Abstract])) OR (Health Mobile*[Title/Abstract])) OR (mobile health[Title/Abstract])) OR (eHealth[Title/Abstract])) OR (Telehealth[Title/Abstract])) OR (Tele-Referrals[Title/Abstract])) OR (Tele-Referral[Title/Abstract])) OR (Virtual Medicine[Title/Abstract])) OR (Tele-Care[Title/Abstract])) OR (Tele Care[Title/Abstract])) OR (Telecare[Title/Abstract])) OR (Teledermatology[Title/Abstract]))) AND (((("malignant melanomas" [tiab:~3]) OR ("malignant melanoma" [tiab:~3])) OR (malignant melanoma*[Title/Abstract])) OR (melanoma*[Title/Abstract])) OR ("Melanoma"[Mesh]))

2.EMBASE (Ovid)
Date: 20/12/2024

2.1.Search Strategy:

1	diagnosis/
2	prognosis/
3	diagnosis.mp.
4	prognosis.mp.
5	prognoses.mp.
6	Breslow.mp.
7	triage*.mp.
8	acces.mp.
9	(factor adj3 prognostic).mp.
10	(prognostic adj3 factors).mp.
11	(malignant adj3 melanomas).mp.
12	(malignant adj3 melanoma).mp.
13	malignant melanoma.mp.
14	melanoma.mp.
15	melanoma/
16	telemedicine/
17	telemedicine.mp.
18	mHealth.mp.
19	eHealth.mp.
20	Telehealth.mp.
21	tele-referrals.mp.
22	tele-referral.mp.
23	virtual medicine.mp.
24	tele-care.mp.

25 tele care.mp.
26 telecare.mp.
27 teledermatology.mp.
28 1 or 2 or 3 or 4 or 5 or 6 or 7 or 8 or 9 or 10
29 11 or 12 or 13 or 14 or 15
30 16 or 17 or 18 or 19 or 20 or 21 or 22 or 23 or 24 or 25 or 26 or 27
31 28 and 29 and 30
32 limit 31 to "remove medline records"
33 limit 32 to conference abstraxct
34 32 not 33

3. WEB OF SCIENCE

Date: 20/12/2024

Web of Science Search Strategy (v0.1)

Database: All Databases

Entitlements:

- WOS: 1900 to 2024
- CCC: 1998 to 2009
- DIIDW: 1980 to 2009
- GRANTS: 1953 to 2024
- KJD: 1980 to 2024
- MEDLINE: 1950 to 2024
- PPRN: 1991 to 2024
- PQDT: 1637 to 2024
- SCIELO: 2002 to 2024

Searches:

1: (TS=(diagnosis))

2: (TS=(diagnoses))

3: (TS=(prognosis))

4: (TS=(prognoses))

5: (TS=(breslow))

6: (TS=(factor NEAR/3 prognostic))

7: (TS=(prognostic NEAR/3 factors))

8: (TS=(triage*))

9: (TS=(access))

10: (#1 OR #2 OR #3 OR #4 OR #5 OR #6 OR #7 OR #8 OR #9)

11: (TS=(telemedicine))

12: (TS=(mHealth))

13: (TS=(health mobile*))

14: (TS=(mobile health))

15: (TS=(eHealth))

16: (TS=(Telehealth))

17: (TS=(Tele-Referrals))

18: (TS=(Tele-referral))

19: (TS=(Virtual Medicine))

20: (TS=(Tele-Care))

21: (TS=(Tele Care))

22: (TS=(Telecare))

23: (TS=(Teledermatology))

24: (#11 OR #12 OR #13 OR #14 OR #15 OR #16 OR #17 OR #18 OR #19 OR #20 OR #21 OR #22 OR #23)

25: (TS=(malignant NEAR/3 melanomas))

26: (TS=(malignant NEAR/3 melanoma))

27: (TS=(malignant NEAR/3 melanoma*))

28: (TS=(melanoma*))

29: (#25 OR #26 OR #27 OR #28)

30: (#10 AND #24 AND #29)

4.SCOPUS
Date: 20/12/2024

4.1.Search Strategy:

(TITLE-ABS-KEY(diagnosis) OR TITLE-ABS-KEY(diagnoses) OR TITLE-ABS-KEY(prognosis) OR TITLE-ABS-KEY(prognoses) OR TITLE-ABS-KEY(breslow) OR TITLE-ABS-KEY(factor AND prognostic) OR TITLE-ABS-KEY(prognostic AND factors) OR TITLE-ABS-KEY(triage*) OR TITLE-ABS-KEY(access)) AND (TITLE-ABS-KEY(teledermatology) OR TITLE-ABS-KEY(telecare) OR TITLE-ABS-KEY(tele AND care) OR TITLE-ABS-KEY(tele-care) OR TITLE-ABS-KEY(virtual AND medicine) OR TITLE-ABS-KEY(tele-referrals) OR TITLE-ABS-KEY(tele-referral) OR TITLE-ABS-KEY(tele AND referral) OR TITLE-ABS-KEY(telehealth) OR

TITLE-ABS-KEY(ehealth) OR TITLE-ABS-KEY(mobile AND health) OR TITLE-ABS-KEY(health AND mobile*) OR TITLE-ABS-KEY(mhealth) OR TITLE-ABS-KEY(telemedicine)) AND (TITLE-ABS-KEY(melanoma*) OR TITLE-ABS-KEY(malignant AND melanoma*) OR TITLE-ABS-KEY(malignant AND melanoma) OR TITLE-ABS-KEY(malignant AND melanomas))

Supplementary Material S2: Risk of Bias Assessments for Each Included Study

Risk of bias was assessed using the Joanna Briggs Institute (JBI) Critical Appraisal Checklists, with each study matched to the most appropriate tool according to its design. Studies evaluating diagnostic accuracy (e.g., sensitivity and specificity of teledermatology or teledermoscopy in melanoma detection) were assessed using the JBI checklist for diagnostic test accuracy studies. Observational studies with an analytical aim (cross-sectional, cohort or quasi-experimental) were appraised using the corresponding JBI checklists. All completed checklists are provided as supplementary files.

The level of risk of bias (low, moderate or high) was graded based on the number and importance of checklist items fulfilled:

- **Low risk of bias** was assigned when most or all items were fulfilled, and any unmet criteria were minor and unlikely to impact the main outcomes.
- **Moderate risk of bias** was assigned when some items were not fulfilled—particularly those related to confounding, measurement validity, or follow-up—and these could plausibly influence the results.
- **High risk of bias** was assigned when several key domains were unmet or unclear, including lack of defined inclusion criteria, inappropriate or missing outcome measures, or major risk of confounding without adjustment.

For studies that did not meet the inclusion criteria of any standard JBI category—such as discrete choice experiments or descriptive studies without clinical outcomes—risk of bias was assessed narratively.

- **Spinks et al. (2015)** conducted a discrete choice experiment (DCE) to explore consumer preferences regarding teledermoscopy. Although not compatible with standard JBI tools, the study applied a clear theoretical framework, defined relevant attributes, and used appropriate statistical models to analyse participant preferences. Its methodological quality was considered acceptable.
- **Horsham et al. (2016)** was a purely descriptive cross-sectional survey assessing public attitudes towards an integrated skin cancer screening model using teledermatology. No comparisons or clinical outcomes were included. The study followed a transparent methodology, with structured questions and appropriate reporting, and was thus narratively appraised.
- **Kirtava et al. (2016)** presented a retrospective descriptive evaluation of a mobile phone image-based teledermatology system, focusing on image quality and diagnostic agreement. As there was no comparative analysis or defined clinical outcomes, it was not eligible for JBI appraisal and was narratively assessed based on data completeness and clarity of reporting.
- **Horsham et al. (2020)** reported results from a user satisfaction and technology acceptance survey embedded within a randomised controlled trial of mobile teledermoscopy. The article did not report clinical outcomes or test accuracy, and therefore could not be assessed using conventional JBI tools. Its structured design and appropriate statistical analysis justified a narrative appraisal.

All four studies were considered methodologically sound for their descriptive purposes and included in the review. Their limitations were taken into account in the interpretation of results but did not justify exclusion.

JBI CRITICAL APPRAISAL CHECKLIST FOR DIAGNOSTIC TEST ACCURACY STUDIES

Reviewer María López-Pardo Rico Date 17th July 2025

Author Tan et al. Year 2010 Record Number 1

	Yes	No	Unclear	Not applicable
1. Was a consecutive or random sample of patients enrolled?	X			
2. Was a case control design avoided?	X			
3. Did the study avoid inappropriate exclusions?	X			
4. Were the index test results interpreted without knowledge of the results of the reference standard?	X			
5. If a threshold was used, was it pre-specified?				X
6. Is the reference standard likely to correctly classify the target condition?	X			
7. Were the reference standard results interpreted without knowledge of the results of the index test?	X			
8. Was there an appropriate interval between index test and reference standard?	X			
9. Did all patients receive the same reference standard?	X			
10. Were all patients included in the analysis?	X			

Overall appraisal: Include [X] Exclude [ ] Seek further info [ ]

Comments (Including reason for exclusion) Well-designed prospective study with consecutive patient inclusion and appropriate reference standard. The same dermatologists evaluated both the index test (teledermoscopy) and the reference test (face-to-face), but measures such as anonymization and a 4-week washout period were implemented to minimise recall bias. Histopathological confirmation was used when available. Overall, the risk of bias is low.

© JBI, 2020. All rights reserved. JBI grants use of these tools for research purposes only. All other enquiries should be sent to jbisynthesis@adelaide.edu.au.

JBI CRITICAL APPRAISAL CHECKLIST FOR DIAGNOSTIC TEST ACCURACY STUDIES

Reviewer María López-Pardo Rico Date 17th July 2025

Author Wolf et al. Year 2013 Record Number 2

	Yes	No	Unclear	Not applicable
1. Was a consecutive or random sample of patients enrolled?	☐	☒	☐	☐
2. Was a case control design avoided?	☐	☒	☐	☐
3. Did the study avoid inappropriate exclusions?	☒	☐	☐	☐
4. Were the index test results interpreted without knowledge of the results of the reference standard?	☒	☐	☐	☐
5. If a threshold was used, was it pre-specified?	☐	☐	☐	☒
6. Is the reference standard likely to correctly classify the target condition?	☒	☐	☐	☐
7. Were the reference standard results interpreted without knowledge of the results of the index test?	☒	☐	☐	☐
8. Was there an appropriate interval between index test and reference standard?	☒	☐	☐	☐
9. Did all patients receive the same reference standard?	☒	☐	☐	☐
10. Were all patients included in the analysis?	☐	☒	☐	☐

Overall appraisal: Include ☒ Exclude ☐ Seek further info ☐

Comments (Including reason for exclusion) Case-control study using pre-selected images with histological confirmation. Although index test results were interpreted blinded to the reference standard and the reference test was appropriate, the use of a case-control design and exclusion of unevaluable images introduce a high risk of selection and spectrum bias. Included in the review, but considered at high risk of bias.

© JBI, 2020. All rights reserved. JBI grants use of these tools for research purposes only. All other enquiries should be sent to jbisynthesis@adelaide.edu.au.

JBI CRITICAL APPRAISAL CHECKLIST FOR DIAGNOSTIC TEST ACCURACY STUDIES

Reviewer María López-Pardo Rico Date 17th July 2025

Author Congalton et al. Year 2015 Record Number 3

	Yes	No	Unclear	Not applicable
1. Was a consecutive or random sample of patients enrolled?	☒	☐	☐	☐
2. Was a case control design avoided?	☒	☐	☐	☐
3. Did the study avoid inappropriate exclusions?	☒	☐	☐	☐
4. Were the index test results interpreted without knowledge of the results of the reference standard?	☒	☐	☐	☐
5. If a threshold was used, was it pre-specified?	☐	☐	☐	☒
6. Is the reference standard likely to correctly classify the target condition?	☒	☐	☐	☐
7. Were the reference standard results interpreted without knowledge of the results of the index test?	☒	☐	☐	☐
8. Was there an appropriate interval between index test and reference standard?	☒	☐	☐	☐
9. Did all patients receive the same reference standard?	☐	☒	☐	☐
10. Were all patients included in the analysis?	☐	☒	☐	☐

Overall appraisal: Include ☒ Exclude ☐ Seek further info ☐

Comments (Including reason for exclusion) Prospective study with consecutive patient inclusion and use of an appropriate reference standard (histopathology when available). The index test was interpreted blinded to the reference, and pre-specified diagnostic thresholds were applied. However, not all lesions underwent histological confirmation, and a small proportion of patients were not included in the final analysis due to missed excisions or no-show. Overall, moderate risk of bias.

© JBI, 2020. All rights reserved. JBI grants use of these tools for research purposes only. All other enquiries should be sent to jbisynthesis@adelaide.edu.au.

JBI CRITICAL APPRAISAL CHECKLIST FOR DIAGNOSTIC TEST ACCURACY STUDIES

Reviewer María López-Pardo Rico Date 17th July 2025

Author Cazzaniga et al. Year 2019 Record Number 4

	Yes	No	Unclear	Not applicable
1. Was a consecutive or random sample of patients enrolled?	☐	☒	☐	☐
2. Was a case control design avoided?	☒	☐	☐	☐
3. Did the study avoid inappropriate exclusions?	☒	☐	☐	☐
4. Were the index test results interpreted without knowledge of the results of the reference standard?	☒	☐	☐	☐
5. If a threshold was used, was it pre-specified?	☐	☐	☐	☒
6. Is the reference standard likely to correctly classify the target condition?	☒	☐	☐	☐
7. Were the reference standard results interpreted without knowledge of the results of the index test?	☒	☐	☐	☐
8. Was there an appropriate interval between index test and reference standard?	☒	☐	☐	☐
9. Did all patients receive the same reference standard?	☐	☒	☐	☐
10. Were all patients included in the analysis?	☐	☒	☐	☐

Overall appraisal: Include ☒ Exclude ☐ Seek further info ☐

Comments (Including reason for exclusion) Prospective validity study embedded in a public awareness campaign. The index test (remote dermatologist assessment) and the reference standard (face-to-face clinical evaluation and biopsy when indicated) were independently performed. Although all participants received the same reference standard and analysis was complete, the sample was self-selected from a population outreach initiative, which may introduce selection bias. Overall, moderate risk of bias.

© JBI, 2020. All rights reserved. JBI grants use of these tools for research purposes only. All other enquiries should be sent to jbisynthesis@adelaide.edu.au.

JBI CRITICAL APPRAISAL CHECKLIST FOR DIAGNOSTIC TEST ACCURACY STUDIES

Reviewer: María López-Pardo Rico Date: 17th July 2025

Author: Jahn et al. Year: 2022 Record Number: 5

	Yes	No	Unclear	Not applicable
1. Was a consecutive or random sample of patients enrolled?	X			
2. Was a case control design avoided?	X			
3. Did the study avoid inappropriate exclusions?	X			
4. Were the index test results interpreted without knowledge of the results of the reference standard?	X			
5. If a threshold was used, was it pre-specified?				X
6. Is the reference standard likely to correctly classify the target condition?	X			
7. Were the reference standard results interpreted without knowledge of the results of the index test?	X			
8. Was there an appropriate interval between index test and reference standard?	X			
9. Did all patients receive the same reference standard?	X			
10. Were all patients included in the analysis?	X			

Overall appraisal: Include [X] Exclude [ ] Seek further info [ ]

Comments (Including reason for exclusion) Well-designed prospective diagnostic accuracy study comparing teledermatology to face-to-face consultation. Consecutive patient inclusion, blinded assessment of index and reference tests, and use of dermoscopy and biopsy as appropriate. All patients received the same reference standard.
Overall, low risk of bias.

© JBI, 2020. All rights reserved. JBI grants use of these tools for research purposes only. All other enquiries should be sent to jbisynthesis@adelaide.edu.au.

JBI CRITICAL APPRAISAL CHECKLIST FOR DIAGNOSTIC TEST ACCURACY STUDIES

Reviewer María López-Pardo Rico Date 17th July 2025

Author Jobbágy et al. Year 2022 Record Number 6

	Yes	No	Unclear	Not applicable
1. Was a consecutive or random sample of patients enrolled?	☐	☒	☐	☐
2. Was a case control design avoided?	☐	☒	☐	☐
3. Did the study avoid inappropriate exclusions?	☒	☐	☐	☐
4. Were the index test results interpreted without knowledge of the results of the reference standard?	☒	☐	☐	☐
5. If a threshold was used, was it pre-specified?	☐	☐	☐	☒
6. Is the reference standard likely to correctly classify the target condition?	☒	☐	☐	☐
7. Were the reference standard results interpreted without knowledge of the results of the index test?	☒	☐	☐	☐
8. Was there an appropriate interval between index test and reference standard?	☒	☐	☐	☐
9. Did all patients receive the same reference standard?	☒	☐	☐	☐
10. Were all patients included in the analysis?	☒	☐	☐	☐

Overall appraisal: Include ☒ Exclude ☐ Seek further info ☐

Comments (Including reason for exclusion) Case-control diagnostic accuracy study using pre-selected histologically confirmed lesions. Although all patients received the same reference standard and index test evaluation was blinded, the artificial composition of the sample (30 melanomas and 70 benign lesions) introduces a high risk of selection and spectrum bias.

© JBI, 2020. All rights reserved. JBI grants use of these tools for research purposes only. All other enquiries should be sent to jbisynthesis@adelaide.edu.au.

JBI CRITICAL APPRAISAL CHECKLIST FOR DIAGNOSTIC TEST ACCURACY STUDIES

Reviewer María López-Pardo Rico Date 17th July 2025

Author Fazil Jaber et al. Year 2023 Record Number 7

	Yes	No	Unclear	Not applicable
1. Was a consecutive or random sample of patients enrolled?	☐	☒	☐	☐
2. Was a case control design avoided?	☒	☐	☐	☐
3. Did the study avoid inappropriate exclusions?	☒	☐	☐	☐
4. Were the index test results interpreted without knowledge of the results of the reference standard?	☒	☐	☐	☐
5. If a threshold was used, was it pre-specified?	☐	☐	☐	☒
6. Is the reference standard likely to correctly classify the target condition?	☒	☐	☐	☐
7. Were the reference standard results interpreted without knowledge of the results of the index test?	☒	☐	☐	☐
8. Was there an appropriate interval between index test and reference standard?	☒	☐	☐	☐
9. Did all patients receive the same reference standard?	☒	☐	☐	☐
10. Were all patients included in the analysis?	☒	☐	☐	☐

Overall appraisal: Include ☒ Exclude ☐ Seek further info ☐

Comments (Including reason for exclusion) Retrospective cross-sectional study comparing TD and FTF in two different patient groups. All lesions were histologically confirmed. Index test interpretation was blinded, and inappropriate exclusions were avoided. However, use of distinct patient groups introduces risk of confounding.
Moderate risk of bias.

© JBI, 2020. All rights reserved. JBI grants use of these tools for research purposes only. All other enquiries should be sent to jbisynthesis@adelaide.edu.au.

JBI CRITICAL APPRAISAL CHECKLIST FOR DIAGNOSTIC TEST ACCURACY STUDIES

Reviewer María López-Pardo Rico Date 17th July 2025

Author Koop et al. Year 2023 Record Number 8

	Yes	No	Unclear	Not applicable
1. Was a consecutive or random sample of patients enrolled?	☒	☐	☐	☐
2. Was a case control design avoided?	☒	☐	☐	☐
3. Did the study avoid inappropriate exclusions?	☒	☐	☐	☐
4. Were the index test results interpreted without knowledge of the results of the reference standard?	☒	☐	☐	☐
5. If a threshold was used, was it pre-specified?	☐	☐	☐	☒
6. Is the reference standard likely to correctly classify the target condition?	☒	☐	☐	☐
7. Were the reference standard results interpreted without knowledge of the results of the index test?	☒	☐	☐	☐
8. Was there an appropriate interval between index test and reference standard?	☒	☐	☐	☐
9. Did all patients receive the same reference standard?	☐	☒	☐	☐
10. Were all patients included in the analysis?	☒	☐	☐	☐

Overall appraisal: Include ☒ Exclude ☐ Seek further info ☐

Comments (Including reason for exclusion) Prospective real-life screening study with consecutive inclusion. Teledermatology assessment was performed prior to the reference standard. Some lesions were confirmed by biopsy, others by clinical follow-up, introducing potential verification bias.
Moderate risk of bias.

© JBI, 2020. All rights reserved. JBI grants use of these tools for research purposes only. All other enquiries should be sent to jbisynthesis@adelaide.edu.au.

JBI CRITICAL APPRAISAL CHECKLIST FOR DIAGNOSTIC TEST ACCURACY STUDIES

Reviewer: María López-Pardo Rico   Date: 17th July 2025

Author: Gafoor et al.   Year: 2024   Record Number: 9

	Yes	No	Unclear	Not applicable
1. Was a consecutive or random sample of patients enrolled?	☐	☒	☐	☐
2. Was a case control design avoided?	☐	☒	☐	☐
3. Did the study avoid inappropriate exclusions?	☒	☐	☐	☐
4. Were the index test results interpreted without knowledge of the results of the reference standard?	☒	☐	☐	☐
5. If a threshold was used, was it pre-specified?	☐	☐	☐	☒
6. Is the reference standard likely to correctly classify the target condition?	☒	☐	☐	☐
7. Were the reference standard results interpreted without knowledge of the results of the index test?	☒	☐	☐	☐
8. Was there an appropriate interval between index test and reference standard?	☒	☐	☐	☐
9. Did all patients receive the same reference standard?	☐	☒	☐	☐
10. Were all patients included in the analysis?	☒	☐	☐	☐

Overall appraisal: Include ☒ Exclude ☐ Seek further info ☐

Comments (Including reason for exclusion) Retrospective service evaluation using a case-enriched sample of patients already attending dermatology clinics. Blinded assessment was performed using patient-captured TD images. Not all lesions were confirmed histologically. Case-control design and verification bias present.
High risk of bias.

© JBI, 2020. All rights reserved. JBI grants use of these tools for research purposes only. All other enquiries should be sent to jbisynthesis@adelaide.edu.au.

JBI CRITICAL APPRAISAL CHECKLIST FOR DIAGNOSTIC TEST ACCURACY STUDIES

Reviewer María López-Pardo Rico Date 17th July 2025

Author Zazo et al. Year 2024 Record Number 10

	Yes	No	Unclear	Not applicable
1. Was a consecutive or random sample of patients enrolled?	X			
2. Was a case control design avoided?	X			
3. Did the study avoid inappropriate exclusions?	X			
4. Were the index test results interpreted without knowledge of the results of the reference standard?	X			
5. If a threshold was used, was it pre-specified?				X
6. Is the reference standard likely to correctly classify the target condition?	X			
7. Were the reference standard results interpreted without knowledge of the results of the index test?	X			
8. Was there an appropriate interval between index test and reference standard?	X			
9. Did all patients receive the same reference standard?	X			
10. Were all patients included in the analysis?	X			

Overall appraisal: Include [X] Exclude [ ] Seek further info [ ]

Comments (Including reason for exclusion) Retrospective cross-sectional study including all histologically confirmed melanomas in one region during 2019. TD was performed prior to diagnosis, and interpretation was blinded. All lesions were confirmed histologically. Low risk of bias.

© JBI, 2020. All rights reserved. JBI grants use of these tools for research purposes only. All other enquiries should be sent to jbisynthesis@adelaide.edu.au.

JBI CRITICAL APPRAISAL CHECKLIST FOR ANALYTICAL CROSS SECTIONAL STUDIES

Reviewer María López-Pardo Rico Date 17th July 2025

Author Ferrándiz et al Year 2012 Record Number 11

	Yes	No	Unclear	Not applicable
1. Were the criteria for inclusion in the sample clearly defined?	X	☐	☐	☐
2. Were the study subjects and the setting described in detail?	X	☐	☐	☐
3. Was the exposure measured in a valid and reliable way?	X	☐	☐	☐
4. Were objective, standard criteria used for measurement of the condition?	X	☐	☐	☐
5. Were confounding factors identified?	☐	X	☐	☐
6. Were strategies to deal with confounding factors stated?	☐	X	☐	☐
7. Were the outcomes measured in a valid and reliable way?	X	☐	☐	☐
8. Was appropriate statistical analysis used?	X	☐	☐	☐

Overall appraisal: Include X Exclude ☐ Seek further info ☐

Comments (Including reason for exclusion) Retrospective analytical cross-sectional study comparing Breslow thickness in TD vs non-TD referred melanomas. Valid outcome measures and appropriate statistical analysis, but no identification or adjustment for confounders. Moderate risk of bias.

© JBI, 2020. All rights reserved. JBI grants use of these tools for research purposes only. All other enquiries should be sent to jbisynthesis@adelaide.edu.au.

JBI CRITICAL APPRAISAL CHECKLIST FOR ANALYTICAL CROSS SECTIONAL STUDIES

Reviewer María López-Pardo Rico Date 17th July 2025

Author Karavan et al Year 2013 Record Number 12

	Yes	No	Unclear	Not applicable
1. Were the criteria for inclusion in the sample clearly defined?	☒	☐	☐	☐
2. Were the study subjects and the setting described in detail?	☒	☐	☐	☐
3. Was the exposure measured in a valid and reliable way?	☒	☐	☐	☐
4. Were objective, standard criteria used for measurement of the condition?	☒	☐	☐	☐
5. Were confounding factors identified?	☐	☒	☐	☐
6. Were strategies to deal with confounding factors stated?	☐	☒	☐	☐
7. Were the outcomes measured in a valid and reliable way?	☒	☐	☐	☐
8. Was appropriate statistical analysis used?	☒	☐	☐	☐

Overall appraisal: Include ☒ Exclude ☐ Seek further info ☐

Comments (Including reason for exclusion) Retrospective analytical cross-sectional study comparing melanoma characteristics in TD vs non-TD groups. Outcome measures were valid and analysis appropriate, but no identification or adjustment for confounding variables. Moderate risk of bias.

© JBI, 2020. All rights reserved. JBI grants use of these tools for research purposes only. All other enquiries should be sent to jbisynthesis@adelaide.edu.au.

JBI CRITICAL APPRAISAL CHECKLIST FOR COHORT STUDIES

Reviewer María López-Pardo Rico Date 17th July 2025

Author Börve et al Year 2015 Record Number 13

	Yes	No	Unclear	Not applicable
1. Were the two groups similar and recruited from the same population?	☒	☐	☐	☐
2. Were the exposures measured similarly to assign people to both exposed and unexposed groups?	☒	☐	☐	☐
3. Was the exposure measured in a valid and reliable way?	☒	☐	☐	☐
4. Were confounding factors identified?	☐	☒	☐	☐
5. Were strategies to deal with confounding factors stated?	☐	☒	☐	☐
6. Were the groups/participants free of the outcome at the start of the study (or at the moment of exposure)?	☒	☐	☐	☐
7. Were the outcomes measured in a valid and reliable way?	☒	☐	☐	☐
8. Was the follow up time reported and sufficient to be long enough for outcomes to occur?	☒	☐	☐	☐
9. Was follow up complete, and if not, were the reasons to loss to follow up described and explored?	☒	☐	☐	☐
10. Were strategies to address incomplete follow up utilized?	☒	☐	☐	☐
11. Was appropriate statistical analysis used?	☒	☐	☐	☐

Overall appraisal: Include ☒ Exclude ☐ Seek further info ☐

Comments (Including reason for exclusion) Prospective cohort study comparing TD-based referrals versus traditional care. Exposure and outcomes were clearly defined and measured reliably. However, no identification or adjustment for confounding variables was performed. Moderate risk of bias.

© JBI, 2020. All rights reserved. JBI grants use of these tools for research purposes only. All other enquiries should be sent to jbisynthesis@adelaide.edu.au.

RoB Assessor: María López-Pardo Rico	Date of Appraisal: 17th July 2025	Record Number: 14
Study Author: Teague et al.	Study Title: Virtual lesion clinic: Evaluation of a teledermatology triage system for referrals in South Australia	Study Year: 2022

Internal Validity		Choice - Comments/Justification	Yes	No	Unclear	N/A
**Bias related to temporal precedence**						
1	Is it clear in the study what is the "cause" and what is the "effect" (i.e. there is no confusion about which variable comes first)?	The study clearly defines the intervention (Virtual Lesion Clinic implementation) and the outcomes (e.g., time to assessment, treatment, Breslow thickness).	☒	☐	☐	☐
**Bias related to selection and allocation**						
2	Was there a control group?	The study compared outcomes between a control group (traditional referrals) and an intervention group (VLC referrals).	☒	☐	☐	☐
**Bias related to confounding factors**						
3	Were participants included in any comparisons similar?	Both groups consisted of patients referred to dermatology for skin lesion assessment in the same healthcare region.	☒	☐	☐	☐
**Bias related to administration of intervention/exposure**						
4	Were the participants included in any comparisons receiving similar treatment/care, other than the exposure or intervention of interest?	All patients were assessed for suspected skin cancer; only the referral pathway differed (VLC vs traditional).	☒	☐	☐	☐

Bias related to assessment, detection and measurement of the outcome

5	Were there multiple measurements of the outcome, both pre and post the intervention/exposure?		Yes	No	Unclear	N/A
	**Outcome 1** Time to assessment	The study compared VLC and traditional care as two independent groups; there were no pre- and post-intervention measurements within the same group.	☐	☒	☐	☐
	**Outcome 2** Time to treatment	No before-after comparison within a single cohort; the outcome was measured once per patient, post-referral.	☐	☒	☐	☐
	**Outcome 3** Surgical/biopsy rate	Data on surgical intervention was collected retrospectively after the referral; there were no repeated measures.	☐	☒	☐	☐
	**Outcome 4** Breslow thickness	Breslow was measured only at diagnosis and compared between groups, not across time within individuals.	☐	☒	☐	☐
	**Outcome 5**		☐	☐	☐	☐
	**Outcome 6**		☐	☐	☐	☐
	**Outcome 7**		☐	☐	☐	☐

6	Were the outcomes of participants included in any comparisons measured in the same way?		Yes	No	Unclear	N/A
	**Outcome 1** Time to assessment	Time was consistently defined as the interval from referral to initial specialist review, measured using the same administrative data across both groups.	☒	☐	☐	☐
	**Outcome 2** Time to treatment	Time to treatment was extracted from clinical records using the same method for VLC and traditional care groups.	☒	☐	☐	☐
	**Outcome 3** Surgical/biopsy rate	All procedures were identified via hospital records using the same coding and documentation system.	☒	☐	☐	☐
	**Outcome 4** Breslow thickness	Histopathological Breslow thickness was obtained from pathology reports with identical protocols across groups.	☒	☐	☐	☐
	**Outcome 5**		☐	☐	☐	☐
	**Outcome 6**		☐	☐	☐	☐

			Yes	No	Unclear	N/A
	**Outcome 7**		☐	☐	☐	☐

7	**Were outcomes measured in a reliable way?**		**Yes**	**No**	**Unclear**	**N/A**
	**Outcome 1** Time to assessment	Dates of referral and specialist review were recorded in the electronic referral and appointment systems, ensuring objective and reliable measurement.	☒	☐	☐	☐
	**Outcome 2** Time to treatment	Treatment dates were obtained from hospital records, which are routinely documented and reliable.	☒	☐	☐	☐
	**Outcome 3** Surgical/biopsy rate	Surgical and biopsy procedures were captured through standardised clinical documentation and coding.	☒	☐	☐	☐
	**Outcome 4** Breslow thickness	Breslow was measured by dermatopathologists using standard histopathological techniques, ensuring high reliability.	☒	☐	☐	☐
	**Outcome 5**		☐	☐	☐	☐
	**Outcome 6**		☐	☐	☐	☐
	**Outcome 7**		☐	☐	☐	☐

Bias related to participant retention

8	**Was follow-up complete and if not, were differences between groups in terms of their follow-up adequately described and analyzed?**					
	**Outcome 1** Time to assessment		**Yes**	**No**	**Unclear**	**N/A**
	Result 1	All referred patients included in the analysis had recorded specialist review dates; there was no loss to follow-up for this outcome.	☒	☐	☐	☐
	Result 2		☐	☐	☐	☐

		Yes	No	Unclear	N/A
Result 3		☐	☐	☐	☐
**Outcome 2** Time to treatment		**Yes**	**No**	**Unclear**	**N/A**
Result 1	Treatment data were available for all relevant patients; the study does not report missing data for this outcome.	☒	☐	☐	☐
Result 2		☐	☐	☐	☐
Result 3		☐	☐	☐	☐
**Outcome 3** Surgical/biopsy rate		**Yes**	**No**	**Unclear**	**N/A**
Result 1	Surgical outcomes were available for all included cases; no differential loss to follow-up was described.	☒	☐	☐	☐
Result 2		☐	☐	☐	☐
Result 3		☐	☐	☐	☐
**Outcome 4** Breslow thickness		**Yes**	**No**	**Unclear**	**N/A**
Result 1	Breslow thickness was reported for all confirmed melanomas; no missing data were indicated.	☒	☐	☐	☐
Result 2		☐	☐	☐	☐
Result 3		☐	☐	☐	☐
**Outcome 5**		**Yes**	**No**	**Unclear**	**N/A**
Result 1		☐	☐	☐	☐
Result 2		☐	☐	☐	☐
Result 3		☐	☐	☐	☐

Outcome 6		Yes	No	Unclear	N/A
Result 1		☐	☐	☐	☐
Result 2		☐	☐	☐	☐
Result 3		☐	☐	☐	☐
**Outcome 7**		**Yes**	**No**	**Unclear**	**N/A**
Result 1		☐	☐	☐	☐
Result 2		☐	☐	☐	☐
Result 3		☐	☐	☐	☐

Statistical Conclusion Validity

9	Was appropriate statistical analysis used?					
	**Outcome 1** Time to assessment		**Yes**	**No**	**Unclear**	**N/A**
	Result 1	Time intervals were analysed using appropriate statistical comparisons (e.g., medians, interquartile ranges); non-parametric tests were used when needed.	☒	☐	☐	☐
	Result 2		☐	☐	☐	☐
	Result 3		☐	☐	☐	☐
	**Outcome 2** Time to treatment		**Yes**	**No**	**Unclear**	**N/A**
	Result 1	The analysis applied suitable statistical tests to compare treatment delays between groups.	☒	☐	☐	☐

			Yes	No	Unclear	N/A
	Result 2		☐	☐	☐	☐
	Result 3		☐	☐	☐	☐
**Outcome 3**	Surgical/biopsy rate		**Yes**	**No**	**Unclear**	**N/A**
	Result 1	Differences in proportions were assessed with appropriate categorical tests (e.g., chi-square or Fisher's exact).	☒	☐	☐	☐
	Result 2		☐	☐	☐	☐
	Result 3		☐	☐	☐	☐
**Outcome 4**	Breslow thickness		**Yes**	**No**	**Unclear**	**N/A**
	Result 1	Breslow thickness was compared using valid methods for continuous variables (e.g., Mann–Whitney U test); the statistical analysis was sound.	☒	☐	☐	☐
	Result 2		☐	☐	☐	☐
	Result 3		☐	☐	☐	☐
**Outcome 5**			**Yes**	**No**	**Unclear**	**N/A**
	Result 1		☐	☐	☐	☐
	Result 2		☐	☐	☐	☐
	Result 3		☐	☐	☐	☐
**Outcome 6**			**Yes**	**No**	**Unclear**	**N/A**
	Result 1		☐	☐	☐	☐
	Result 2		☐	☐	☐	☐

Result 3		☐	☐	☐	☐
**Outcome 7**		**Yes**	**No**	**Unclear**	**N/A**
Result 1		☐	☐	☐	☐
Result 2		☐	☐	☐	☐
Result 3		☐	☐	☐	☐

Overall appraisal: **Include:** ☑ **Exclude:** ☐ **Seek Further Info:** ☐

Comments:

Teague et al., 2022 is a retrospective quasi-experimental study comparing outcomes between a virtual lesion clinic (VLC) and traditional care. The cause-effect relationship and g roup comparability were clearly established. All outcomes were measured consistently, reliably, and using appropriate statistical methods. However, the study lacked pre-post measurements within the same group, and no confounding adjustment was performed. Overall, the study was judged to have a low to moderate risk of bias.

© JBI, 2022. All rights reserved. JBI grants use of these tools for research purposes only. All other enquiries should be sent to jbisynthesis@adelaide.edu.au

JBI CRITICAL APPRAISAL CHECKLIST FOR ANALYTICAL CROSS SECTIONAL STUDIES

Reviewer: María López-Pardo Rico Date: 17th July 2025

Author: Jaklitsch et al Year: 2024 Record Number: 15

	Yes	No	Unclear	Not applicable
1. Were the criteria for inclusion in the sample clearly defined?	X	☐	☐	☐
2. Were the study subjects and the setting described in detail?	X	☐	☐	☐
3. Was the exposure measured in a valid and reliable way?	X	☐	☐	☐
4. Were objective, standard criteria used for measurement of the condition?	X	☐	☐	☐
5. Were confounding factors identified?	X	☐	☐	☐
6. Were strategies to deal with confounding factors stated?	X	☐	☐	☐
7. Were the outcomes measured in a valid and reliable way?	X	☐	☐	☐
8. Was appropriate statistical analysis used?	X	☐	☐	☐

Overall appraisal: Include X Exclude ☐ Seek further info ☐

Comments (Including reason for exclusion) Retrospective analytical cross-sectional study using EMR data to compare Breslow thickness in melanoma diagnosed via teledermatology vs face-to-face visits. Inclusion criteria were clearly defined, exposures and outcomes were measured using reliable sources, and appropriate multivariable analyses were applied. Low risk of bias.

© JBI, 2020. All rights reserved. JBI grants use of these tools for research purposes only. All other enquiries should be sent to jbisynthesis@adelaide.edu.au.

JBI CRITICAL APPRAISAL CHECKLIST FOR COHORT STUDIES

Reviewer María López-Pardo Rico Date 17th July 2025

Author May et al Year 2008 Record Number 16

	Yes	No	Unclear	Not applicable
1. Were the two groups similar and recruited from the same population?	X			
2. Were the exposures measured similarly to assign people to both exposed and unexposed groups?	X			
3. Was the exposure measured in a valid and reliable way?	X			
4. Were confounding factors identified?		X		
5. Were strategies to deal with confounding factors stated?		X		
6. Were the groups/participants free of the outcome at the start of the study (or at the moment of exposure)?	X			
7. Were the outcomes measured in a valid and reliable way?	X			
8. Was the follow up time reported and sufficient to be long enough for outcomes to occur?	X			
9. Was follow up complete, and if not, were the reasons to loss to follow up described and explored?	X			
10. Were strategies to address incomplete follow up utilized?				X
11. Was appropriate statistical analysis used?	X			

Overall appraisal: Include [X] Exclude [ ] Seek further info [ ]

Comments (Including reason for exclusion) Prospective cohort study comparing access to dermatology and treatment timelines between patients referred via teledermatology and conventional pathways. Exposure and outcomes were reliably measured, and follow-up was complete. However, no confounding factors were identified or adjusted for. Moderate risk of bias.

© JBI, 2020. All rights reserved. JBI grants use of these tools for research purposes only. All other enquiries should be sent to jbisynthesis@adelaide.edu.au.

JBI CRITICAL APPRAISAL CHECKLIST FOR ANALYTICAL CROSS SECTIONAL STUDIES

Reviewer María López-Pardo Rico Date 17th July 2025

Author Dahlén Gyllencreutz et al Year 2017 Record Number 17

	Yes	No	Unclear	Not applicable
1. Were the criteria for inclusion in the sample clearly defined?	X			
2. Were the study subjects and the setting described in detail?	X			
3. Was the exposure measured in a valid and reliable way?	X			
4. Were objective, standard criteria used for measurement of the condition?	X			
5. Were confounding factors identified?		X		
6. Were strategies to deal with confounding factors stated?		X		
7. Were the outcomes measured in a valid and reliable way?	X			
8. Was appropriate statistical analysis used?	X			

Overall appraisal: Include ☒ Exclude ☐ Seek further info ☐

Comments (Including reason for exclusion) Cross-sectional study comparing time to specialist consultation between teledermoscopy and paper referrals in melanoma cases. The exposure and outcome were measured reliably, but confounding factors were not identified or adjusted for. Moderate risk of bias.

© JBI, 2020. All rights reserved. JBI grants use of these tools for research purposes only. All other enquiries should be sent to jbisynthesis@adelaide.edu.au.

JBI CRITICAL APPRAISAL CHECKLIST FOR ANALYTICAL CROSS SECTIONAL STUDIES

Reviewer: María López-Pardo Rico Date: 17th July 2025

Author: Teoh & Oakley et al Year: 2022 Record Number: 18

	Yes	No	Unclear	Not applicable
1. Were the criteria for inclusion in the sample clearly defined?	X	☐	☐	☐
2. Were the study subjects and the setting described in detail?	X	☐	☐	☐
3. Was the exposure measured in a valid and reliable way?	X	☐	☐	☐
4. Were objective, standard criteria used for measurement of the condition?	X	☐	☐	☐
5. Were confounding factors identified?	☐	X	☐	☐
6. Were strategies to deal with confounding factors stated?	☐	X	☐	☐
7. Were the outcomes measured in a valid and reliable way?	X	☐	☐	☐
8. Was appropriate statistical analysis used?	X	☐	☐	☐

Overall appraisal: Include [X] Exclude ☐ Seek further info ☐

Comments (Including reason for exclusion) Retrospective cross-sectional study reviewing a 9-year teledermoscopy service in New Zealand. Inclusion criteria and outcomes were clearly defined and measured reliably. However, no confounding factors were identified or adjusted for. Moderate risk of bias.

© JBI, 2020. All rights reserved. JBI grants use of these tools for research purposes only. All other enquiries should be sent to jbisynthesis@adelaide.edu.au.

JBI CRITICAL APPRAISAL CHECKLIST FOR ANALYTICAL CROSS SECTIONAL STUDIES

Reviewer María López-Pardo Rico Date 17th July 2025

Author Bouton et al Year 2024 Record Number 19

	Yes	No	Unclear	Not applicable
1. Were the criteria for inclusion in the sample clearly defined?	☒	☐	☐	☐
2. Were the study subjects and the setting described in detail?	☒	☐	☐	☐
3. Was the exposure measured in a valid and reliable way?	☒	☐	☐	☐
4. Were objective, standard criteria used for measurement of the condition?	☒	☐	☐	☐
5. Were confounding factors identified?	☐	☒	☐	☐
6. Were strategies to deal with confounding factors stated?	☐	☒	☐	☐
7. Were the outcomes measured in a valid and reliable way?	☒	☐	☐	☐
8. Was appropriate statistical analysis used?	☒	☐	☐	☐

Overall appraisal: Include ☒ Exclude ☐ Seek further info ☐

Comments (Including reason for exclusion) Retrospective cross-sectional study comparing melanoma management timelines between AI-supported teledermatology and conventional pathways. Exposure and outcomes were clearly defined and measured using reliable sources. However, confounding factors were neither identified nor controlled. Moderate risk of bias.

© JBI, 2020. All rights reserved. JBI grants use of these tools for research purposes only. All other enquiries should be sent to jbisynthesis@adelaide.edu.au.

JBI CRITICAL APPRAISAL CHECKLIST FOR COHORT STUDIES

Reviewer María López-Pardo Rico Date 17th July 2025

Author Sahin et al Year 2024 Record Number 20

	Yes	No	Unclear	Not applicable
1. Were the two groups similar and recruited from the same population?	X			
2. Were the exposures measured similarly to assign people to both exposed and unexposed groups?	X			
3. Was the exposure measured in a valid and reliable way?	X			
4. Were confounding factors identified?	X			
5. Were strategies to deal with confounding factors stated?		X		
6. Were the groups/participants free of the outcome at the start of the study (or at the moment of exposure)?	X			
7. Were the outcomes measured in a valid and reliable way?	X			
8. Was the follow up time reported and sufficient to be long enough for outcomes to occur?	X			
9. Was follow up complete, and if not, were the reasons to loss to follow up described and explored?	X			
10. Were strategies to address incomplete follow up utilized?				X
11. Was appropriate statistical analysis used?	X			

Overall appraisal: Include ☒ Exclude ☐ Seek further info ☐

Comments (Including reason for exclusion) Retrospective cohort study comparing melanoma-related outcomes before and after the implementation of teledermatology. Groups were comparable and data collection was reliable. Although potential confounders were acknowledged, they were not statistically adjusted for. Low to moderate risk of bias.

© JBI, 2020. All rights reserved. JBI grants use of these tools for research purposes only. All other enquiries should be sent to jbisynthesis@adelaide.edu.au.

JBI CRITICAL APPRAISAL CHECKLIST FOR ANALYTICAL CROSS SECTIONAL STUDIES

Reviewer María López-Pardo Rico Date 17th July 2025

Author Koh et al Year 2019 Record Number 21

	Yes	No	Unclear	Not applicable
1. Were the criteria for inclusion in the sample clearly defined?	X	☐	☐	☐
2. Were the study subjects and the setting described in detail?	X	☐	☐	☐
3. Was the exposure measured in a valid and reliable way?	X	☐	☐	☐
4. Were objective, standard criteria used for measurement of the condition?	X	☐	☐	☐
5. Were confounding factors identified?	X	☐	☐	☐
6. Were strategies to deal with confounding factors stated?	X	☐	☐	☐
7. Were the outcomes measured in a valid and reliable way?	X	☐	☐	☐
8. Was appropriate statistical analysis used?	X	☐	☐	☐

Overall appraisal: Include X Exclude ☐ Seek further info ☐

Comments (Including reason for exclusion) Cross-sectional survey assessing user satisfaction with a mobile teledermatology app. The study used validated and structured questionnaires, identified and adjusted for potential confounders, and employed appropriate statistical analysis. Low risk of bias.

© JBI, 2020. All rights reserved. JBI grants use of these tools for research purposes only. All other enquiries should be sent to jbisynthesis@adelaide.edu.au.

JBI CRITICAL APPRAISAL CHECKLIST FOR ANALYTICAL CROSS SECTIONAL STUDIES

Reviewer María López-Pardo Rico Date 17th July 2025

Author Chin et al Year 2021 Record Number 22

	Yes	No	Unclear	Not applicable
1. Were the criteria for inclusion in the sample clearly defined?	X			
2. Were the study subjects and the setting described in detail?	X			
3. Was the exposure measured in a valid and reliable way?	X			
4. Were objective, standard criteria used for measurement of the condition?	X			
5. Were confounding factors identified?		X		
6. Were strategies to deal with confounding factors stated?		X		
7. Were the outcomes measured in a valid and reliable way?	X			
8. Was appropriate statistical analysis used?	X			

Overall appraisal: Include [X] Exclude [ ] Seek further info [ ]

Comments (Including reason for exclusion) Cross-sectional survey evaluating patient satisfaction with a teledermatology triage system. The study used structured questionnaires and descriptive analysis but did not identify or adjust for potential confounders. Moderate risk of bias.

© JBI, 2020. All rights reserved. JBI grants use of these tools for research purposes only. All other enquiries should be sent to jbisynthesis@adelaide.edu.au.

JBI CRITICAL APPRAISAL CHECKLIST FOR ANALYTICAL CROSS SECTIONAL STUDIES

Reviewer María López-Pardo Rico Date 17th July 2025

Author Damsin et al Year 2020 Record Number 23

	Yes	No	Unclear	Not applicable
1. Were the criteria for inclusion in the sample clearly defined?	X			
2. Were the study subjects and the setting described in detail?	X			
3. Was the exposure measured in a valid and reliable way?	X			
4. Were objective, standard criteria used for measurement of the condition?	X			
5. Were confounding factors identified?		X		
6. Were strategies to deal with confounding factors stated?		X		
7. Were the outcomes measured in a valid and reliable way?	X			
8. Was appropriate statistical analysis used?	X			

Overall appraisal: Include [X] Exclude [ ] Seek further info [ ]

Comments (Including reason for exclusion) Cross-sectional study reporting preliminary results of a teledermoscopy project in primary care. Reliable data were used to assess diagnostic and timing outcomes, but potential confounders were not identified or adjusted for. Moderate risk of bias.

© JBI, 2020. All rights reserved. JBI grants use of these tools for research purposes only. All other enquiries should be sent to jbisynthesis@adelaide.edu.au.
